# The microbiota regulates murine inflammatory responses to toxin-induced CNS demyelination but has minimal impact on remyelination

**DOI:** 10.1073/pnas.1905787116

**Published:** 2019-11-18

**Authors:** Christopher E. McMurran, Alerie Guzman de la Fuente, Rosana Penalva, Ofra Ben Menachem-Zidon, Yvonne Dombrowski, John Falconer, Ginez A. Gonzalez, Chao Zhao, Fynn N. Krause, Adam M. H. Young, Julian L. Griffin, Clare A. Jones, Claire Hollins, Markus M. Heimesaat, Denise C. Fitzgerald, Robin J. M. Franklin

**Affiliations:** ^a^Wellcome–MRC Stem Cell Institute, University of Cambridge, CB2 0AW Cambridge, United Kingdom;; ^b^Wellcome–Wolfson Institute for Experimental Medicine, Queen’s University Belfast, BT9 7BL Belfast, United Kingdom;; ^c^Hadassah Human Embryonic Stem Cell Research Center, Goldyne Savad Institute of Gene Therapy, Hadassah-Hebrew University Hospital, 91240 Jerusalem, Israel;; ^d^Department of Biochemistry, University of Cambridge, CB2 1GA Cambridge, United Kingdom;; ^e^Respiratory, Inflammation and Autoimmunity Research, MedImmune Ltd., CB21 6GH Cambridge, United Kingdom;; ^f^Institute for Microbiology, Infectious Diseases and Immunology, Charité–University Medicine Berlin, 14195 Berlin, Germany

**Keywords:** microbiota, remyelination, microglia, macrophage, oligodendrocyte progenitor cell

## Abstract

The microbiota is now recognized as a key influence on the host immune response in the central nervous system (CNS). As such, there has been some progress toward therapies that modulate the microbiota with the aim of limiting immune-mediated demyelination, as occurs in multiple sclerosis. However, remyelination—the regeneration of myelin sheaths—also depends upon an immune response, and the effects that such interventions might have on remyelination have not yet been explored. Here, we show that the inflammatory response during CNS remyelination in mice is modulated by antibiotic or probiotic treatment, as well as in germ-free mice. We also explore the effect of these changes on oligodendrocyte progenitor cell differentiation, which is inhibited by antibiotics but unaffected by our other interventions. These results reveal that high combined doses of oral antibiotics impair oligodendrocyte progenitor cell responses during remyelination and further our understanding of how mammalian regeneration relates to the microbiota.

## Significance

People with multiple sclerosis have a microbiota distinct from healthy controls, and there is growing interest in how these differences might contribute to the onset and progression of CNS autoimmunity. However, the impact that the microbiota may also have on the endogenous regeneration of myelin—remyelination—has not yet been explored. Here we show that inflammatory responses during remyelination depend upon the microbiota, being modulated by antibiotics or probiotics or in germ-free mice. In contrast, these interventions had minimal impact on the activity of oligodendrocyte progenitor cells, with only supratherapeutic doses of antibiotics having an inhibitory effect. Our results suggest that endogenous CNS remyelination is largely resilient to interventions that modify the microbiota.

Our knowledge of the microbiota and its relationship with host immunity, metabolism, and neurobiology has significantly expanded in recent years (1). However, the role of the microbiota in mammalian regeneration remains relatively unexplored. As endogenous tissue regeneration is facilitated by a local immune response (2), it is feasible that intestinal microbes, which modulate the host immune system (3, 4), could help shape the outcome of regeneration in a clinical setting. The gut microbial community can be easily modified using oral antibiotics or probiotics; thus, addressing this question is of great clinical relevance.

Here, we investigate how the intestinal microbiota influences remyelination, the most effective form of regeneration observed in the mammalian central nervous system (CNS) and a promising therapeutic strategy for multiple sclerosis (MS) and other myelin diseases (5). Successful remyelination can bring functional recovery (6, 7) and protect axons from degeneration (8). This success depends on an inflammatory response by microglia and infiltrating macrophages (9) that progresses and resolves appropriately (10, 11). Due to its distance from any epithelial interface with environmental microbes, CNS remyelination is an ideal model to explore systemic influences of the microbiota on regeneration.

The rationale for our studies is based on the close relationship between remyelination and the innate immune response, and, in turn, between the immune system and the microbiota. To create an environment that permits remyelination, endogenous microglia and infiltrating macrophages must clear myelin debris remaining from the disintegrated sheath (12), and also secrete proregenerative factors (10, 11, 13). These roles hinge upon a coordinated immune response to demyelination, failure of which can impair remyelination, as occurs in older animals (14, 15). Meanwhile, there is now a substantial body of evidence linking the microbiota to CNS inflammation across various contexts. For example, germ-free (GF) or antibiotics-treated mice have transcriptionally immature microglia, which have an impaired response to lipopolysaccharide (LPS) or viral stimulation (3), while antibiotic depletion of the microbiota reduces monocyte entry into the brain (4). The immune system is therefore a strong candidate for conveying an influence from the microbiota to regeneration in distant tissues such as the CNS.

There is a growing appreciation of the relationship between the microbiota and MS. Patients with MS have a distinct microbiome compared to healthy controls (16, 17), and this may have a role in disease pathogenesis, given that fecal transplant from patients with MS can provoke immune-mediated demyelination in predisposed GF mice (18). Much focus has centered upon antibiotic or probiotic interventions that aim to limit demyelination in MS and animal models (19–21). However, the effects that such treatments might have on remyelination remain to be elucidated.

Here we use a variety of interventions to alter the murine microbiota, before quantifying inflammation and oligodendrocyte progenitor cell (OPC) activity in response to toxin-induced demyelination. Across antibiotics-treated, probiotic-treated, and GF mice, altering the microbiota was found to modulate the inflammatory response following demyelination. However, no clear relationship emerged between the microbiota, the OPC response to demyelination, and subsequent remyelination.

## Results

### Combined Antibiotics Treatment to Deplete the Microbiota Alters Inflammation following Toxin-Induced Demyelination.

**Fig. 1.**
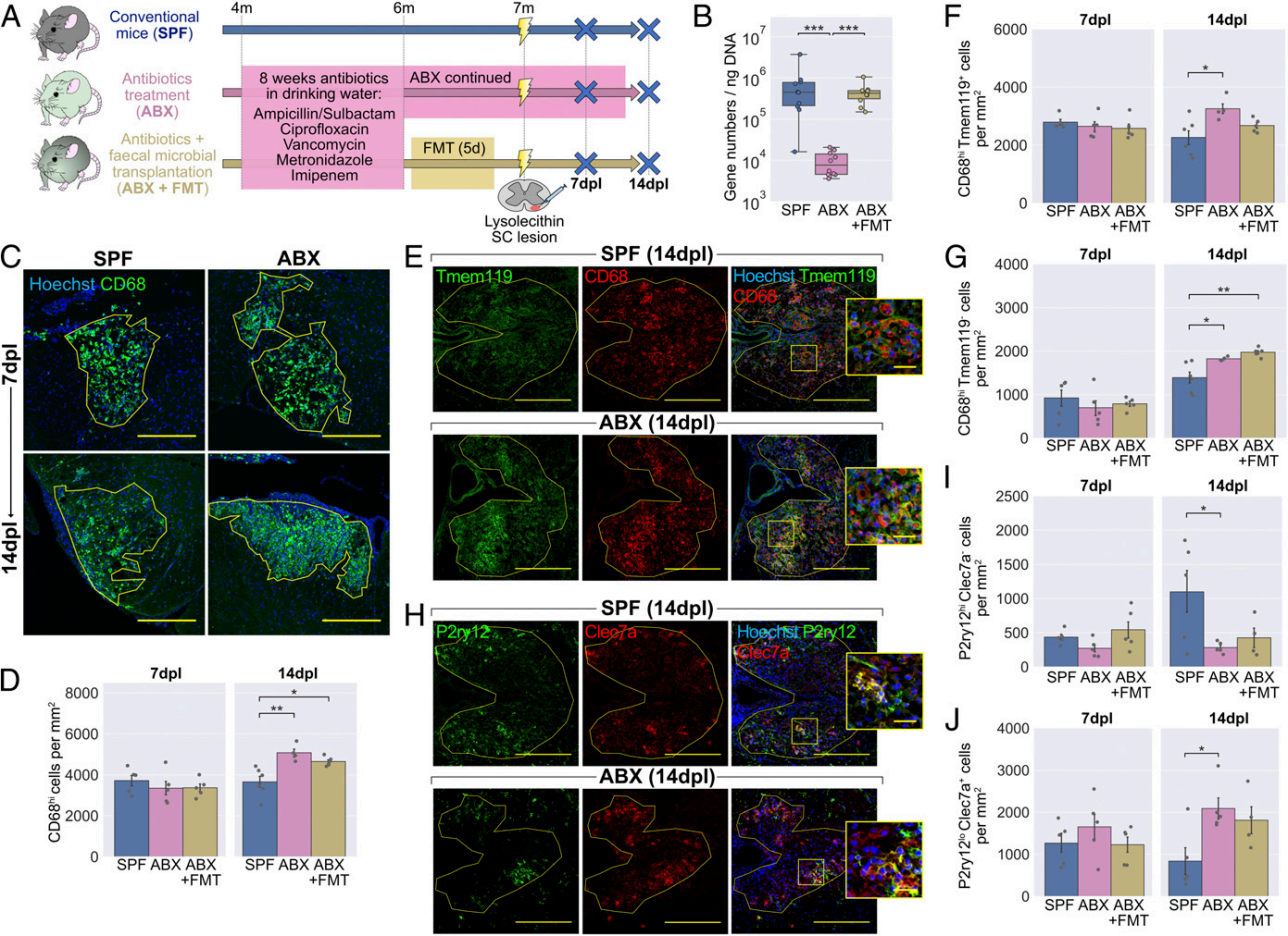
Antibiotics treatment to deplete the microbiota alters the inflammatory response following lysolecithin-mediated demyelination. (*A*) Mice were administered ABX in their drinking water for 2 mo, after which one group received an FMT while another group were continued on ABX. Mice were killed at 7 and 14 d following lysolecithin injection into the ventral white matter of the spinal cord. (*B*) RT-PCR of fecal DNA showing depletion of the microbiota by ABX and return to normal levels with FMT. (*C*) Representative images and (*D*) density of CD68^hi^ activated microglia/macrophages within the lesion boundary (yellow line). (*E*) Representative images and density of (*F*) Tmem119^+^CD68^hi^ microglia-derived and (*G*) Tmem119^−^CD68^hi^ monocyte-derived CD68^hi^ cells within lesions. (*H*) Representative images and density of (*I*) P2ry12^hi^Clec7a^−^ homeostatic and (*J*) P2ry12^lo^Clec7a^+^ degeneration-associated microglia/macrophages within lesions. *Insets* in *E* and *H* are a 3× magnification of the boxed regions. (Scale bars: *C*, 250 µm; *E* and *H*, 200 µm, and *Insets*, 25 µm.) Error bars show mean ± SEM; *P < 0.05, **P < 0.01, ***P < 0.001; in *B*, Kruskal−Wallis with Dunn’s post hoc test; in *D*, *F*, *G*, *I*, and *J*, 1-way ANOVA with Tukey HSD post hoc test, *n* = 4 to 6 mice.

To explore whether extensive microbial depletion would affect remyelination, C57BL/6 mice were administered broad-spectrum antibiotics (ABX) in their drinking water for 8 wk (Fig. 1*A*). This combination of ampicillin/sulbactam, ciprofloxacin, vancomycin, metronidazole, and imipenem has previously been shown to cause depletion of the microbiota (4), which we confirmed by quantitative RT-PCR of fecal DNA (Fig. 1*B*). Demyelination was then induced by focal injection of lysolecithin into the ventral white matter of the spinal cord.

To investigate baseline differences in microglia following ABX treatment, areas of unlesioned white matter were stained with an antibody to Iba1. Microglial morphology, which correlates with activity within a given tissue (3, 22), was then analyzed (*SI Appendix*, Fig. S1*A*). While a principal component analysis based on a number of morphological features showed clear separation between microglia from gray and white matter, there were no differences detected in microglia of unlesioned white matter following ABX treatment (*SI Appendix*, Fig. S1 *B* and *C*). This approach gave a useful indication of baseline microglial function, although we cannot exclude subtle differences that may have been resolved with more sensitive tools such as 3-dimensional morphometric analysis or RNA sequencing (3).

The inflammatory response within lesions was examined at 7 and 14 d postlesion (dpl) by staining for CD68. While CD68 is constitutively expressed at low levels in homeostatic microglia (23), we counted highly CD68-positive cells (CD68^hi^)—a population comprising activated microglia as well as infiltrating macrophages. At 7 dpl, the density of CD68^hi^ cells was similar between ABX-treated mice and specific pathogen-free (SPF) controls (Fig. 1 *C* and *D*), with no difference in lesion size (*SI Appendix*, Fig. S1*D*). However, at 14 dpl, the total density of CD68^hi^ activated microglia/macrophages was increased in lesions of mice that had received ABX treatment (Fig. 1 *C* and *D*). To determine the reversibility of this effect, ABX-treated mice received a fecal microbial transplant (FMT) from SPF mice by oral gavage to reconstitute their microbiota. With FMT treatment, CD68^hi^ cell density remained higher than in SPF controls (Fig. 1*D*). We then asked whether the differences were driven primarily by microglia or infiltrating monocytes, by costaining for CD68 with Tmem119, a stable marker for microglia (24). We observed that both CD68^hi^Tmem119^+^ microglia and CD68^hi^Tmem119^−^ monocyte-derived macrophages were increased in number with ABX treatment (Fig. 1 *E*–*G*).

Subpopulations of microglia/macrophages are known to have distinct effects in remyelination (11). We used antibodies to P2ry12 and Clec7a to identify homeostatic (P2ry12^hi^Clec7a^−^) and degeneration-associated (P2ry12^lo^Clec7a^+^) phenotypes (25, 26). At 14 dpl, as well as a higher total number of CD68^hi^ cells, lesions of ABX-treated mice had fewer P2ry12^hi^Clec7a^−^ homeostatic microglia (Fig. 1 *H* and *I*) and more of the P2ry12^lo^Clec7a^+^ degeneration-associated phenotype (Fig. 1 *H* and *J*) than in SPF controls. We also identified a population within all lesions that expressed both Clec7a and high levels of P2ry12 (*SI Appendix*, Fig. S1*O*). Staining for a panel of other microglia/macrophage markers with recognized roles in remyelination (*SI Appendix*, Fig. S1 *E*–*L*) revealed further differences between ABX-treated mice and controls. At the 7-dpl time point, ABX-treated mice had fewer CD68^hi^ cells expressing MHC class II (MHC II; *SI Appendix*, Fig. S1 *E* and *F*) or arginase-1 (Arg1; *SI Appendix*, Fig. S1 *G* and *H*), with variable reversal following FMT.

Taken together, these results indicate that ABX treatment results in dysregulated CNS inflammation following demyelination. Specifically, we observed an increase in total CD68^hi^ cells from 7 to 14 dpl in ABX-treated groups, not seen among SPF controls. Coupled with this, MHC II and Arg1 expression by microglia/macrophages in the ABX-treated groups were reduced at 7 dpl, and, at the later time point, there was a preponderance of the degeneration-associated microglia/macrophages with reduced numbers of P2ry12^hi^Clec7a^−^ homeostatic microglia. Thus, our microbial depletion model was dominated by a delayed but aggressive proinflammatory innate immune response.

### Combined Antibiotics Treatment Impairs Myelin Debris Clearance and OPC Differentiation.

Central to CNS remyelination is the generation of new oligodendrocytes to restore the myelin sheath. These derive from endogenous OPCs, which migrate to lesions, proliferate, and subsequently differentiate into oligodendrocytes (5, 27). Defective differentiation from OPC to oligodendrocyte is commonly the bottleneck at which remyelination fails in human lesions and animal models (28–30). Microglia and infiltrating macrophages promote remyelination through the clearance of myelin debris, which blocks OPC differentiation in culture (31) and impairs remyelination in demyelinated lesions (12). We investigated whether the dysregulated inflammatory response following ABX treatment would be associated with reduced myelin debris clearance.

**Fig. 2.**
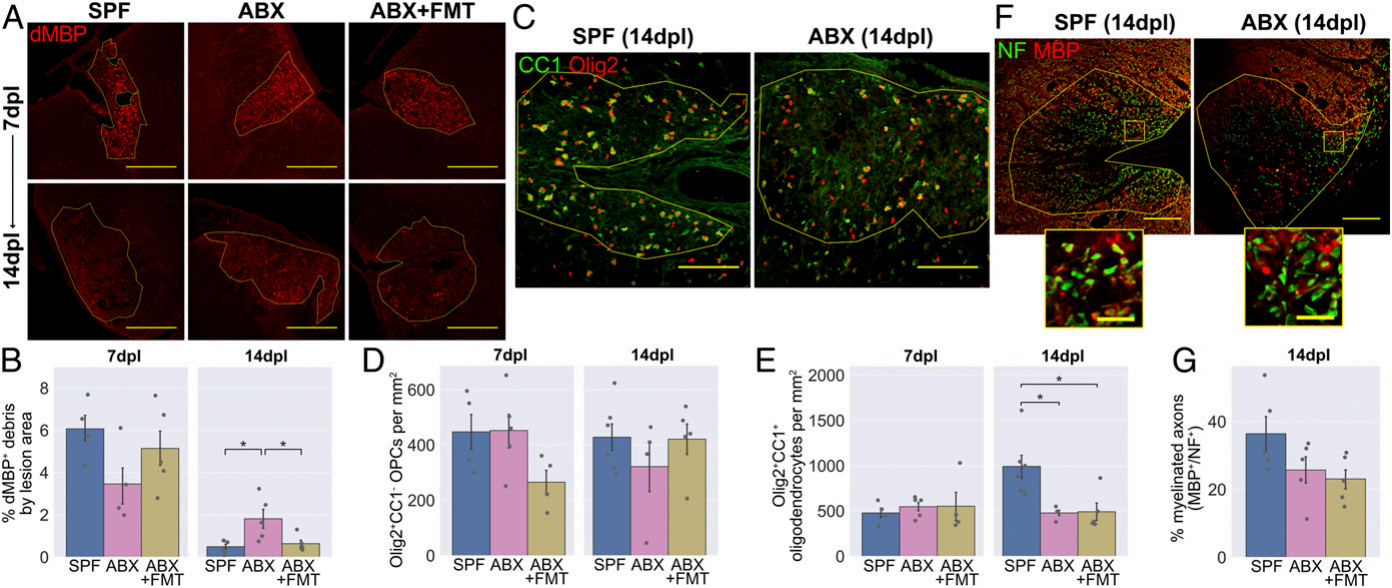
Fewer new oligodendrocytes are generated in the lesions of ABX-treated mice. (*A*) Representative images and (*B*) area of dMBP^+^ myelin debris within lesions. (*C*) Representative images and densities of (*D*) Olig2^+^CC1^−^ OPCs and (*E*) Olig2^+^CC1^+^ oligodendrocytes within lesions. (*F*) Representative images and (*G*) quantification of NF^+^ axons and MBP^+^ myelin sheaths within lesions. *Insets* in *F* are a 10× magnification of the boxed regions. (Scale bars: *A*, 250 µm; *C*, 100 µm; *F*, 100 µm, and *Insets*, 20 µm.) Error bars show mean ± SEM; *P < 0.05; 1-way ANOVA with Tukey HSD post hoc test, *n* = 4 to 6 mice.

Lesions were stained with an antibody for a degraded epitope of myelin basic protein (dMBP), which becomes exposed in myelin debris (32) (Fig. 2 *A* and *B*). At 7 dpl, antibiotics did not affect the quantity of myelin debris in the lesion area. By 14 dpl, there had been an expected reduction in myelin debris in the SPF controls; however, this was less pronounced among the ABX-treated group, with more debris persisting at the later time point. The effect was reversed in the FMT group, which had a similar dMBP^+^ area to controls.

We next explored how the OPC response would be affected by ABX treatment, using the nuclear transcription factor Olig2 to label cells of the oligodendrocyte lineage and using CC1 to distinguish differentiated oligodendrocytes (CC1^+^) from OPCs (CC1^−^). ABX did not affect the total number of OPCs present (Fig. 2 *C* and *D*), nor the number of proliferating OPCs identified by Ki67 staining (*SI Appendix*, Fig. S2). However, there were fewer differentiated oligodendrocytes in the lesions of ABX-treated mice at 14 dpl, suggesting a defect in OPC differentiation (Fig. 2 *C* and *E*). This was not restored by FMT in the ABX-treated group. Finally, we quantified myelinated axons at 14 dpl by counting neurofilament^+^ axons surrounded by a ring of MBP^+^ myelin. There were no significant differences between the groups (*P* = 0.10; Fig. 2 *F* and *G*).

In summary, following demyelination in ABX-treated mice, the altered inflammatory response is associated with impaired myelin debris clearance and impaired OPC differentiation. The effects of ABX treatment on remyelinating lesions could feasibly be caused by either ABX-mediated depletion of the microbiota, other off-target effects of this oral ABX regime, or a combination of these mechanisms. Two approaches were taken to explore these possibilities: 1) testing for off-target effects of ABX on relevant CNS cells in culture and 2) investigating remyelination in GF mice, which were reared in a sterile environment and thus were constitutionally devoid of microbes without ABX exposure.

### Combined Antibiotics Treatment Does Not Affect Microglial Phagocytosis or OPC Differentiation In Vitro.

**Fig. 3.**
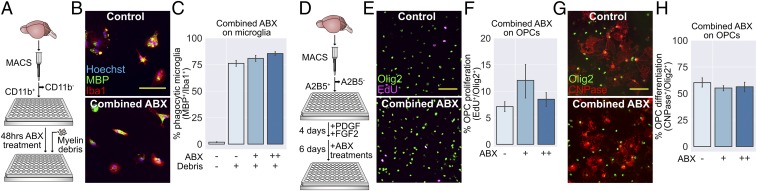
Antibiotics treatment does not affect microglial phagocytosis or OPC responses in vitro. (*A*) Primary microglia were isolated from 3-mo-old mice using MACS for CD11b, cultured for 48 h in the presence of antibiotics, then exposed to myelin debris for 4 h. (*B*) Microglia were stained with an antibody to MBP to visualize myelin uptake. (*C*) Myelin uptake following exposure to combined antibiotics at their estimated CNS concentration (++) or a 10% dose (+) was comparable to control levels (−). (*D*) Primary OPCs were isolated from P6-8 mice using MACS for A2B5. After 4 d, antibiotics were applied, and growth factors were withdrawn to allow differentiation for 6 further days. (*E*–*H*) Cells were stained with an antibody to Olig2 in combination with (*E*) labeling incorporated EdU to visualize proliferation or (*G*) CNPase to visualize differentiation. The antibiotic treatments had no significant effect on OPC (*F*) proliferation or (*H*) differentiation. (Scale bars: *B*, *E*, and *G*, 100 μm.) Error bars show mean ± SEM; paired-samples *t* test with Holm−Bonferroni correction, *n* = 4 to 5 separate experiments.

Some antibiotics are able to penetrate and act directly within the CNS. One example is the broad-spectrum tetracycline, minocycline, which can directly inhibit microglia and influence remyelination (33–35). Four of the antibiotics we administered were considered to have sufficient bioavailability to reach the CNS in significant concentrations: ampicillin, ciprofloxacin, metronidazole, and the β-lactamase inhibitor sulbactam. Available pharmacokinetic data were used to estimate the concentrations these drugs would reach in the murine CNS (*SI Appendix*, Fig. S3*A*), which were then applied to adult primary murine microglial cultures for 48 h (Fig. 3*A*). Following ABX treatment, microglia were exposed to myelin debris, and phagocytic uptake was quantified after 4 h. None of the ABX individually (*SI Appendix*, Fig. S3 *C* and *D*), nor in combination (Fig. 3 *B* and *C*), directly inhibited myelin phagocytosis by microglia. To confirm that we were not overlooking any dynamic changes by analyzing a single time point, we also performed a time course of phagocytosis in the presence of the combination treatment and similarly found no difference from control conditions (*SI Appendix*, Fig. S3*B*).

It is also possible that ABX can act directly on OPCs to inhibit differentiation in lesions of mice receiving oral ABX. To test this, primary cultures of murine OPCs were treated with ABX, and the effects on differentiation and proliferation were determined by immunocytochemistry after 6 d of differentiation conditions (Fig. 3*D*). ABX treatment had no effect on OPC proliferation as measured by 5-ethynyl-2′-deoxyuridine (EdU) incorporation (Fig. 3 *E* and *F*), nor was there a difference in expression of the differentiation marker 2′,3′-cyclic-nucleotide 3′-phosphodiesterase (CNPase) (Fig. 3 *G* and *H*). Similarly, applying each antibiotic alone had no effect on these parameters (*SI Appendix*, Fig. S3 *E*–*H*).

### GF Mice Have an Altered Inflammatory Response during Remyelination.

**Fig. 4.**
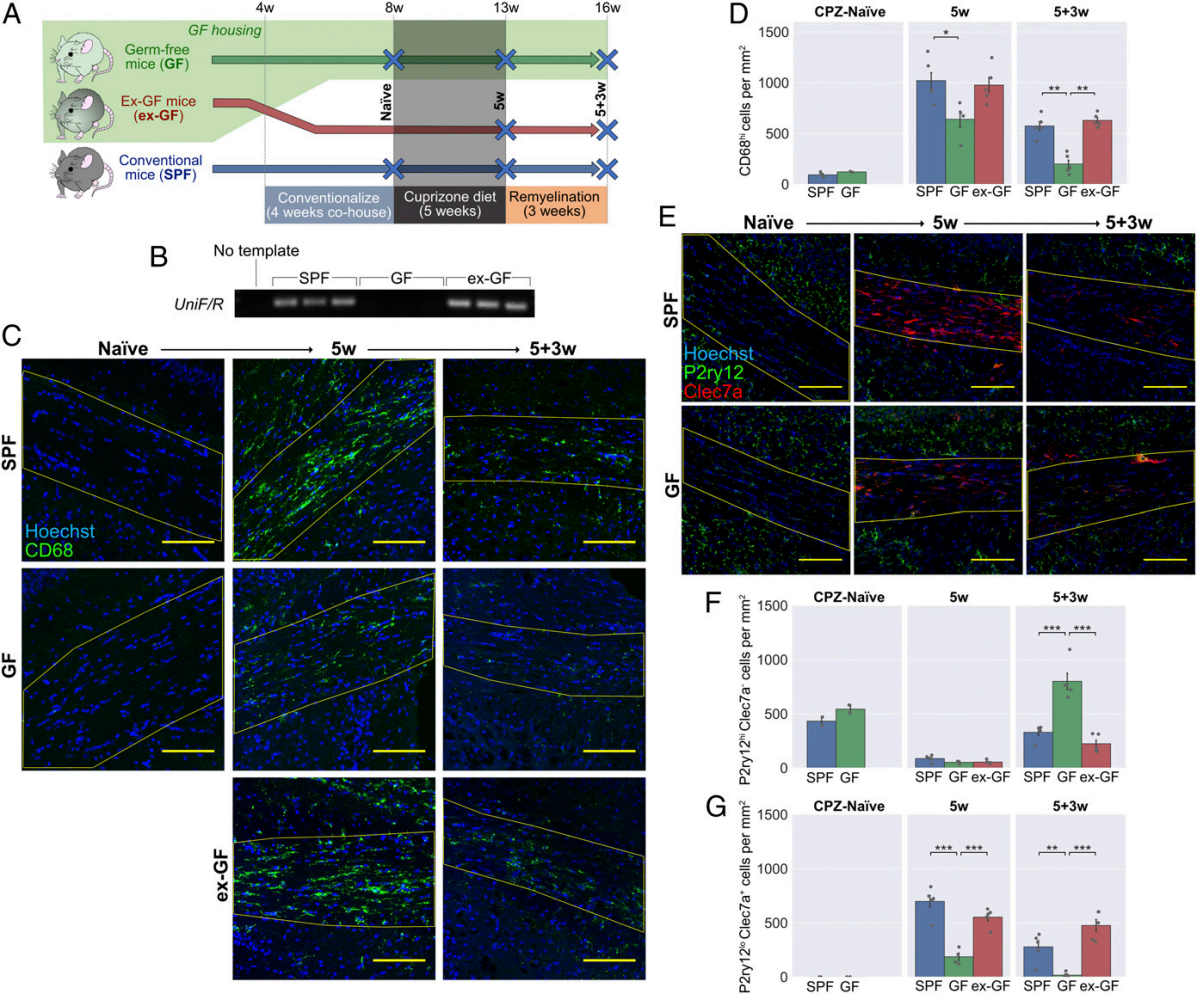
GF mice have an altered inflammatory response following cuprizone-mediated demyelination. (*A*) GF mice and SPF controls were fed a diet containing 0.2% cuprizone for 5 wk from 8 wk of age. Mice were killed at the end of cuprizone administration, or after 3 wk of return to normal diet. A third group consisted of GF mice that were cohoused with SPF mice from 4 wk of age, becoming colonized with a microbiota after weaning. (*B*) PCR for a universal prokaryotic 16S sequence in fecal DNA, demonstrating absence of this amplicon in GF mice and its presence in the ex-GF group. (*C*) Representative images and (*D*) density of CD68^hi^ activated microglia/macrophages within the corpus callosum (yellow line) in cuprizone-naïve mice, following 5 wk cuprizone exposure (5w) and following a further 3 wk of normal diet (5+3w). (*E*) Representative images and density of (*F*) P2ry12^hi^Clec7a^−^ homeostatic and (*G*) P2ry12^lo^Clec7a^+^ degeneration-associated microglia/macrophages within the corpus callosum. (Scale bars: *C* and *E*, 100 μm.) Error bars show mean ± SEM; *P < 0.05, **P < 0.01, ***P < 0.001; 1-way ANOVA with Tukey HSD post hoc test, *n* = 4 to 5 mice.

The results from cell culture systems suggest that the deficits observed in ABX-treated mice are not caused by direct effects of antibiotics on microglial or OPC functions critical for remyelination. However, antibiotics may have other indirect effects that are difficult to capture in vitro. To determine whether the microbiota can influence remyelination in the absence of ABX exposure, we investigated how remyelination would proceed in GF mice. As surgical lesions are impractical under the constraints of GF husbandry, we employed the cuprizone model, in which demyelination is induced by dietary administration of 0.2% cuprizone over a period of 5 wk (Fig. 4*A*). The sterility of the GF mice was confirmed by fecal PCR for bacterial DNA (Fig. 4*B*) as well as routine in-house screening of animals and isolators using aerobic and anaerobic culture methods and microscopy of fecal smears. An ex-GF group were colonized with a microbiota after weaning, by cohousing with SPF controls, and this allowed us to distinguish developmental effects of lacking a microbiota from those reversible in adulthood. After cuprizone exposure, all mice were returned to a normal diet for a 3-wk period of remyelination.

GF mice had differences in the inflammatory response that accompanied demyelination. The total number of CD68^hi^ activated microglia and monocyte-derived macrophages was reduced in the GF group after the 5-wk cuprizone treatment and remained so 3 wk after cuprizone was withdrawn (Fig. 4 *C* and *D*). To investigate this response in more detail, we quantified homeostatic (P2ry12^hi^Clec7a^−^) and degeneration-associated (P2ry12^lo^Clec7a^+^) phenotypes across the 3 groups (Fig. 4 *E*–*G*). Degeneration-associated microglia/macrophages accumulated in the corpus callosum following cuprizone exposure, but this occurred to a much lesser extent in the GF group (Fig. 4*G*). In contrast, 3 wk after cuprizone withdrawal, GF mice had higher numbers of homeostatic microglia than the other groups (Fig. 4*F*). Numbers of CD68^hi^, P2ry12^hi^Clec7a^−^, and P2ry12^lo^Clec7a^+^ cells were all comparable to SPF controls in the ex-GF group, demonstrating that colonization of GF mice can restore immune responsiveness and delineates an ongoing, dynamic role of the microbiota in the inflammatory response to tissue damage, rather than a critical window during development.

As with the ABX-treated mice, we sought to distinguish microglia and monocyte-derived macrophages. However, we observed that Tmem119 was expressed at very low levels within the corpus callosum following cuprizone exposure (*SI Appendix*, Fig. S4*A*) and thus was less useful as a microglia-specific marker in this context. Instead, we quantified coexpression of Iba1 with P2ry12 (36), which remained detectable (albeit at lower levels in reactive microglia), allowing distinction between Iba1^+^P2ry12^+^ microglia and Iba1^+^P2ry12^−^ peripheral macrophages (*SI Appendix*, Fig. S4 *B*–*E*). Both microglia and monocyte-derived macrophages were reduced in GF mice after 5 wk of cuprizone, while, 3 wk later, the reduced CD68 expression of GF mice was primarily associated with reduced numbers of Iba1^+^P2ry12^−^ peripheral macrophages (*SI Appendix*, Fig. S4 *D* and *E*). We also stained for the same panel of microglia/macrophage markers as tested in the antibiotics experiment. Previously, ABX-treated mice were found to have reduced expression of both MHC II and Arg1 during remyelination (*SI Appendix*, Fig. S1 *E*–*H*). Here, MHC II was similarly diminished in the remyelinating corpus callosum of GF mice, with negligible expression in microglia/macrophages of any of these animals (*SI Appendix*, Fig. S4 *F* and *G*). There was no significant reduction in Arg1, despite a similar trend (*SI Appendix*, Fig. S4 *H* and *I*), nor was there a difference detected in inducible nitric oxide synthase (iNOS) or mannose receptor (MR) expression in GF mice (*SI Appendix*, Fig. S4 *J*–*M*).

The deficits in the immune response of ABX-treated mice were associated with reduced myelin debris clearance after demyelination (Fig. 2 *A* and *B*). A time course of cuprizone feeding in SPF mice showed that extensive dMBP^+^ myelin debris after 3 wk of cuprizone diet is subsequently cleared by 5 wk (*SI Appendix*, Fig. S4*N*). We subsequently compared dMBP staining at our 5-wk time point to determine whether myelin debris clearance was impaired under GF conditions. All groups showed similarly low levels of dMBP staining at 5 wk, indicating that any difference in debris clearance was not persistent beyond 5 wk (*SI Appendix*, Fig. S4*O*).

In summary, we observed that the inflammatory response following demyelination was also altered in GF mice. However, while lesions of ABX-treated mice were characterized by higher density of degeneration-associated microglia/macrophages, the opposite was true for cuprizone-treated GF mice, in which homeostatic microglia dominated the inflammatory response. The changes in GF mice suggest that the microbiota can indeed influence the immune response in the CNS during demyelination and remyelination; however, in this case, there was no associated change in myelin debris clearance.

### GF Mice Have Normal OPC Differentiation during Remyelination.

**Fig. 5.**
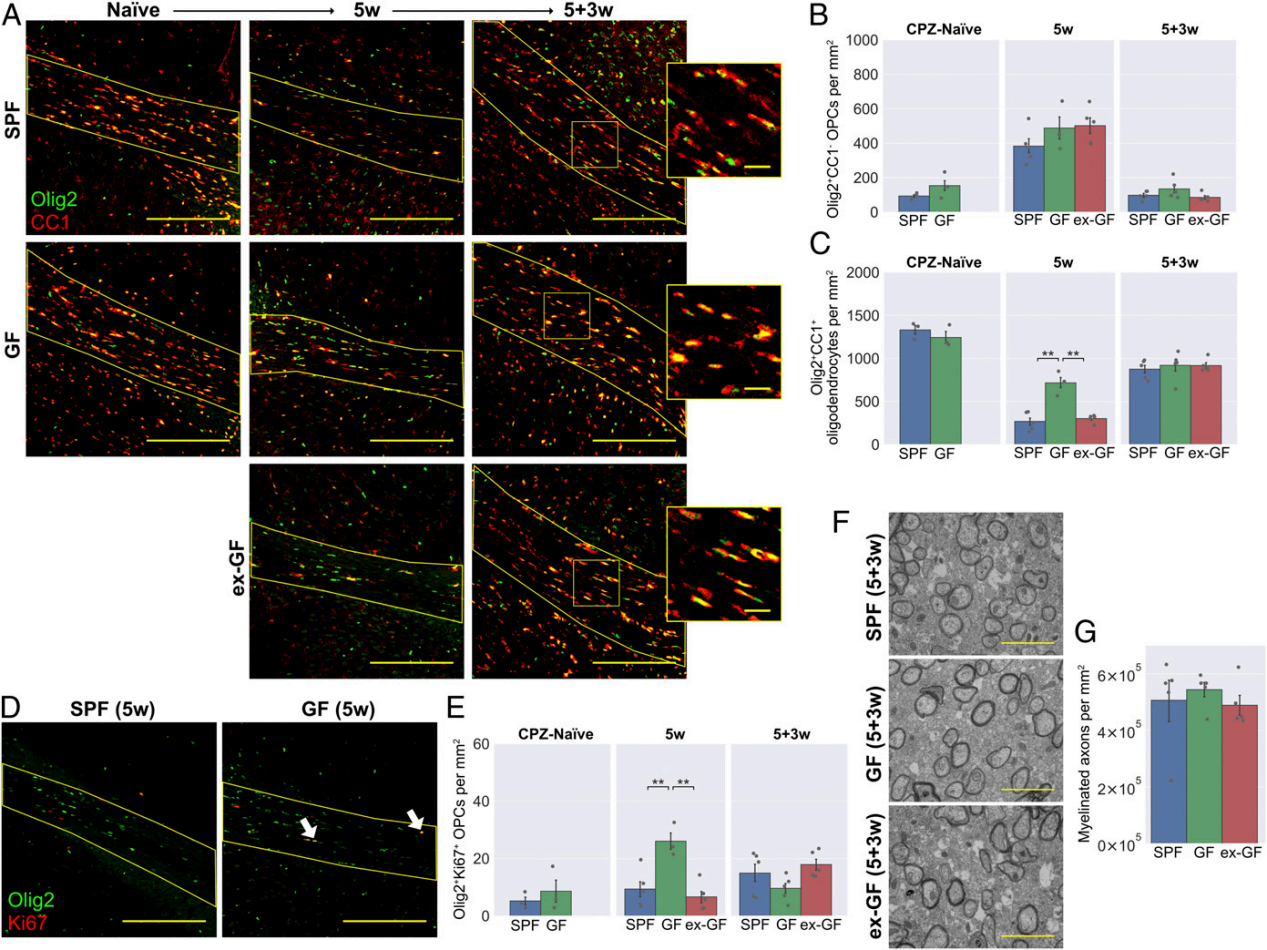
GF mice have reduced oligodendrocyte loss following cuprizone administration, but no difference in OPC differentiation. (*A*) Representative images and density of (*B*) Olig2^+^CC1^−^ OPCs and (*C*) Olig2^+^CC1^+^ mature oligodendrocytes within the corpus callosum in cuprizone-naïve mice, following 5 wk cuprizone exposure (5w) and following a further 3 wk of normal diet (5+3w). *Insets* in *A* are a 2.5× magnification of the boxed regions. (*D*) Representative images and (*E*) density of Olig2^+^Ki67^+^ proliferating OPCs within the corpus callosum. Arrow heads in *D* show representative double-positive cells. (*F*) Representative electron microscopy images and (*G*) quantification of myelinated axons within the corpus callosum 3 wk after cuprizone cessation. (Scale bars: *A*, 200 μm, and *Insets*, 25 μm; *D*, 200 μm; *F*, 2 μm.) Error bars show mean ± SEM; **P < 0.01; 1-way ANOVA, *n* = 3 to 5 mice.

Next, the OPC responses during cuprizone-mediated demyelination and remyelination were examined in GF mice. Prior to cuprizone exposure, GF and SPF mice had comparable numbers of Olig2^+^CC1^−^ OPCs (Fig. 5 *A* and *B*) and Olig2^+^CC1^+^ oligodendrocytes (Fig. 5 *A* and *C*) in the corpus callosum. Following 5-wk cuprizone treatment, there was an 80% reduction in the density of Olig2^+^CC1^+^ oligodendrocytes in the SPF group (Fig. 5*C*). In contrast, the loss of oligodendrocytes after 5 wk of cuprizone was less extensive among the GF group, while the ex-GF group were in line with SPF controls. At this time point, all groups experienced a similar rise in Olig2^+^CC1^−^ OPCs (Fig. 5*B*), consistent with the phase of OPC migration and proliferation known to occur during the first 5 wk of cuprizone administration (37). However, when we specifically quantified Olig2^+^Ki67^+^ proliferative OPCs at 5 wk, this was higher in the GF mice than in the other 2 groups (Fig. 5 *D* and *E*).

Three weeks after cuprizone withdrawal, a time point at which remyelination should be progressing (37, 38), all 3 groups exhibited a fall in OPC counts with a concurrent rise in differentiated oligodendrocytes. Importantly, there were no differences in OPC or oligodendrocyte numbers between the groups at this later time point (Fig. 5 *B* and *C*), suggesting that there was no delay in OPC differentiation during the remyelination of GF mice.

Finally, the degree of remyelination following cuprizone cessation was investigated by transmission electron microscopy (Fig. 5*F*). The corpus callosum of SPF control, GF, and ex-GF mice all exhibited comparable densities of myelinated axons 3 wk after cuprizone treatment was stopped, indicating that remyelination had progressed similarly between groups (Fig. 5*G*). The g-ratios of these axons were also measured and likewise revealed no differences (*SI Appendix*, Fig. S5).

In summary, GF mice were partially resistant to the effects of cuprizone, with less extensive oligodendrocyte loss following 5 wk of dietary administration. This effect could be related to their diminished innate immune response during cuprizone treatment (Fig. 4), given that various studies have demonstrated a role of the immune system, particularly microglia and neutrophils, in mediating cuprizone-induced demyelination (39, 40). The late peak in OPC proliferation observed in the GF group (Fig. 5 *D* and *E*) may similarly be in response to a sluggish innate immune response. However, 3 wk after cuprizone was stopped, the absence of any difference in OPC or oligodendrocyte numbers combined with similar densities of myelinated axons suggest that remyelination itself is unaffected in GF mice.

### Probiotic VSL#3 Can Augment the Innate Immune Response during CNS Remyelination.

**Fig. 6.**
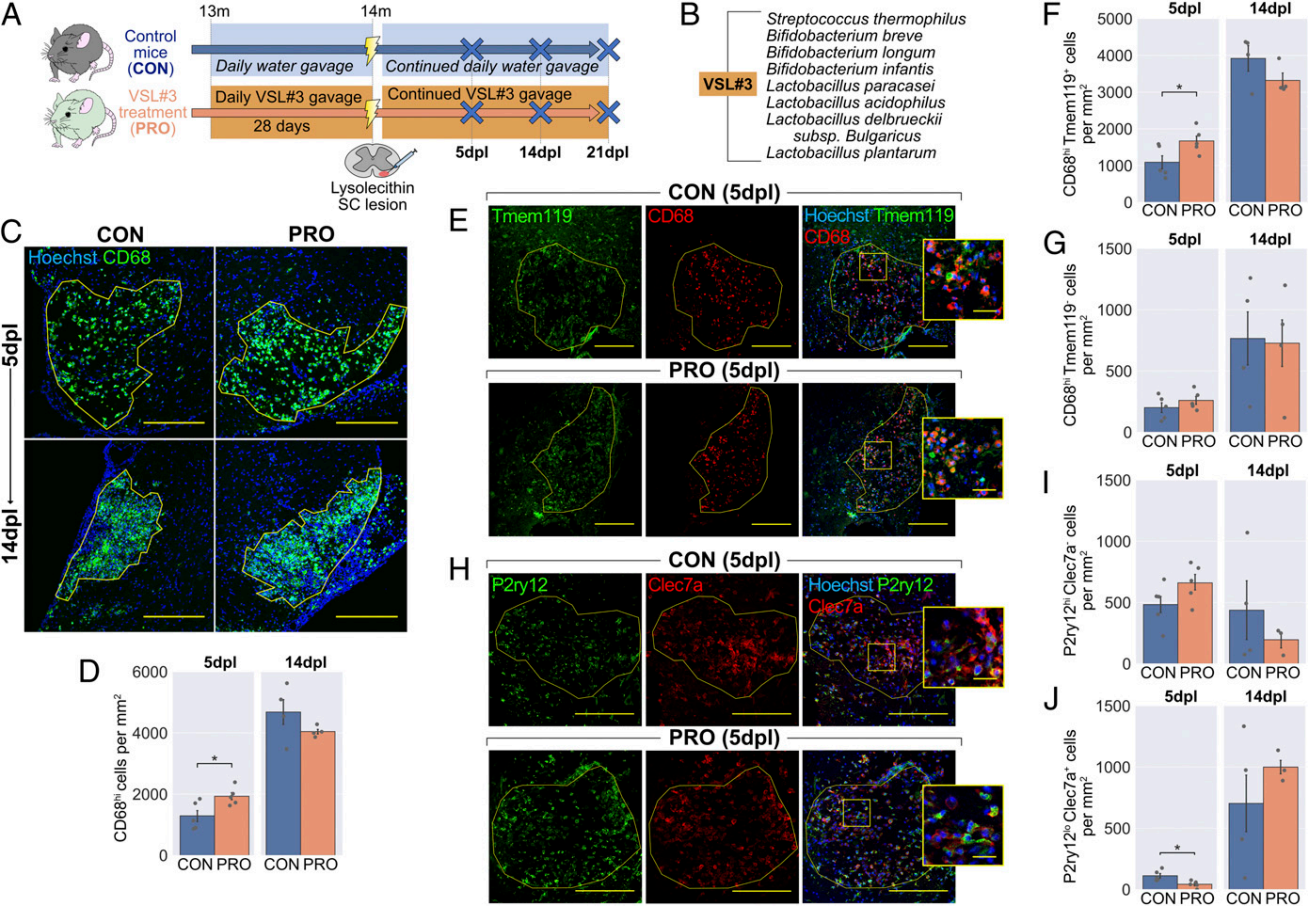
Probiotic VSL#3 enhances the onset of inflammation following demyelination. (*A*) The 13-mo-old mice were administered 1.35 × 10^9^ CFU VSL#3 daily for 1 mo by oral gavage. Mice were killed at 5 and 14 d following lysolecithin injection into the ventral white matter of the spinal cord. (*B*) Constituent strains of VSL#3. (*C*) Representative images and (*D*) density of CD68^hi^ activated microglia/macrophages within lesions. (*E*) Representative images and density of (*F*) Tmem119^+^CD68^hi^ microglia-derived and (*G*) Tmem119^−^CD68^hi^ monocyte-derived CD68^hi^ cells within lesions. (*H*) Representative images and density of (*I*) P2ry12^hi^Clec7a^−^ homeostatic and (*J*) P2ry12^lo^Clec7a^+^ degeneration-associated microglia/macrophages within lesions. Insets in *E* and *H* are a 3× magnification of the boxed regions. (Scale bars: *C*, 250 μm; *E* and *H*, 200 μm, and *Insets*, 25 μm.) Error bars show mean ± SEM; *P < 0.05; Student’s *t* test, *n* = 3 to 5 mice.

Taking the results from the antibiotics and GF experiments, together with in vitro studies, an intact microbiota appears to be necessary for the appropriate inflammatory response during remyelination. However, the effect that microbial depletion has on OPC responses, and thus remyelination itself, remains less certain. We next explored the inverse of this relationship: whether the microbiota can be manipulated therapeutically to enhance appropriate inflammatory responses and subsequently remyelination. For this, we used older mice (aged 14 mo) in which remyelination occurs more slowly with scope for improvement (14, 41). These mice were administered the probiotic VSL#3 by daily gavage for 1 mo, prior to inducing focal demyelination in the spinal cord white matter by lysolecithin injection (Fig. 6*A*).

VSL#3, a freeze-dried formulation of 8 strains of Gram-positive bacteria (Fig. 6*B*), was chosen due to its good survival on transit through the gastrointestinal tract (42) and several characterized therapeutic effects in the CNS (4, 43, 44). Additionally, VSL#3 enhanced concentrations of short-chain fatty acids (SCFAs) in the feces and serum of mice (*SI Appendix*, Fig. S6 *B* and *D*), consistent with a previous study (45). The SCFAs acetate, butyrate, and propionate are microbial metabolites that are depleted in GF mice (*SI Appendix*, Fig. S5 *A* and *C*) and considered key signaling molecules in how the microbiota influence CNS inflammation (3, 46).

Following VSL#3 treatment, there was a mild enhancement in the inflammatory response at 5 dpl, with greater density of CD68^hi^ activated microglia and infiltrating macrophages (Fig. 6 *C* and *D*). We again stained for CD68 in combination with Tmem119 to determine which populations of CD68^hi^ cells were affected. We found that probiotic administration increased numbers of CD68^hi^Tmem119^+^ microglia at 5 dpl (Fig. 6 *E* and *F*), although no difference was detected among CD68^hi^Tmem119^−^ monocyte-derived macrophages (Fig. 6 *E* and *G*). Staining for P2ry12 and Clec7a revealed that, despite the increase in total number of CD68^hi^ cells at 5 dpl, the numbers of P2ry12^lo^Clec7a^+^ degeneration-associated microglia/macrophages were reduced with VSL#3 (Fig. 6 *H*–*J*), suggesting a less detrimental inflammatory environment. Both iNOS and MR were expressed by greater numbers of CD68^hi^ cells in lesions following probiotic treatment at 5 dpl (*SI Appendix*, Fig. S6 *E*–*H*), while 2 markers initially reduced by ABX (MHC II and Arg1) were not significantly enhanced by probiotic treatment (*SI Appendix*, Fig. S6 *I*–*L*).

No differences were detected at 14 dpl, a time point at which debris should be largely cleared and newly differentiated oligodendrocytes initiate remyelination. Thus, although our probiotic augmented the initial inflammatory response at 5 dpl, the effect of this on the subsequent trajectory of inflammation in the lesion was more limited.

### VSL#3 Does Not Improve the Outcome of Remyelination.

**Fig. 7.**
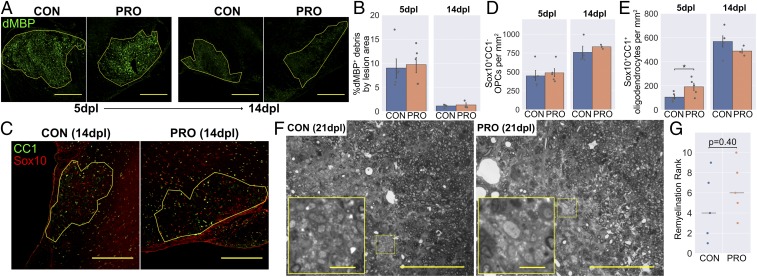
VSL#3 probiotic does not enhance remyelination in aged mice. (*A*) Representative images and (*B*) area of dMBP^+^ myelin debris within lesions. (*C*) Representative images and density of (*D*) Sox10^+^CC1^−^ OPCs and (*E*) Sox10^+^CC1^+^ mature oligodendrocytes within lesions. (*F*) Representative images of toluidine blue-stained resin sections demonstrating persistent demyelination, typical of aged mice (control [CON] rank: 4/10, probiotic [PRO] rank: 3/10). Images are shown in grayscale. *Insets* are a 4× magnification of the boxed regions. (*G*) Remyelination ranks assigned by a blinded assessor, with horizontal lines showing the median for each group. (Scale bars: *A* and *C*, 250 μm; *F*, 100 μm, and *Insets*, 10 μm.) Error bars show mean ± SEM; *P < 0.05; in *B*, *D*, and *E*, Student’s *t* test, *n* = 3 to 5 mice; in *G*, Mann–Whitney *U* test, *n* = 5 mice.

Finally, we assessed whether this inflammatory enhancement during the early stages would contribute to a better outcome in remyelination. In contrast to ABX-treated mice, in which the altered inflammatory response resulted in prolonged presence of myelin debris, myelin debris was not cleared any faster in the lesions of probiotic-treated mice (Fig. 7 *A* and *B*). Consistent with this, there was no difference in the OPC response, with OPC number (Fig. 7 *C* and *D* and *SI Appendix*, Fig. S7*A*), proliferation (*SI Appendix*, Fig. S7 *B* and *C*), and differentiation of new oligodendrocytes at 14 dpl (Fig. 7 *C* and *E*) unchanged by probiotic treatment. There was a small increase in the number of oligodendrocytes present at 5 dpl (Fig. 7*E* and *SI Appendix*, Fig. S7*A*). As this time point is considered too early for significant OPC differentiation to occur, even in young adults (47) and taking into account that no difference was observed at 14 dpl, the peak time for OPC differentiation, this likely reflects a reduction in oligodendrocyte death, rather than enhanced OPC differentiation.

To confirm that there was no therapeutic effect of VSL#3 on remyelination, semithin resin sections were taken at a later time point (21 dpl) and myelin-stained with toluidine blue (Fig. 7*F*). The completion of remyelination was ranked by 2 blinded assessors, neither of whom detected a difference in remyelination between groups (Fig. 7*G*).

Thus, administration of the probiotic VSL#3 promoted a stronger initial inflammatory response in aged mice following demyelination. However, this did not lead to faster clearance of myelin debris or OPC differentiation, and overall remyelination was unchanged compared to control mice.

## Discussion

The aim of these experiments was to explore how the microbiota can influence remyelination in the mammalian CNS. Across all 3 models, the responses of microglia and infiltrating macrophages were modulated by interventions that altered the microbiota. Broadly, this amounted to a dysregulated immune response in ABX-treated mice, and a blunted, more homeostatic-type response in GF mice. In contrast, VSL#3 probiotic treatment enhanced the onset of inflammation while reducing numbers of damage-associated microglia/macrophages. As remyelination depends upon a coordinated immune response (9–11, 48), such changes might be expected to impact upon the OPC activity underlying remyelination. Consistent with this hypothesis, ABX-treated mice had deficits in myelin debris clearance and OPC differentiation. However, these findings were neither replicated in GF mice nor reversed by probiotic administration. Bringing together these findings from different models, it appears that the microbiota shapes the inflammatory response during CNS remyelination, but the effect of this on regenerative responses by OPCs is limited.

We can postulate what the reasons may be for this disparity, in which modulating the microbiota alters CNS inflammatory responses without simultaneous changes in the efficiency of remyelination itself. It could be that the inflammatory changes that our interventions provoked via the microbiota were simply not sufficient to alter remyelination. This may be either a quantitative limit (i.e., the changes in CNS inflammation caused by microbiota modulation were too subtle) or a more qualitative limit (i.e., the specific functions of microglia/macrophages that were affected had minimal effect on remyelination). As new molecular techniques reveal further insights into microglial/macrophage heterogeneity, the specific roles of these different phenotypes during remyelination remain to be fully elucidated (25, 26). Similarly, it cannot be excluded that other microbiota-based interventions might be more fruitful in producing changes in the efficiency of remyelination. However, we believe that our negative result showing a lack of impact on remyelination using GF mice is evidence against such a powerful influence.

In vitro, ABX did not directly influence microglial phagocytosis or OPC differentiation, although other indirect effects of ABX are difficult to exclude. Indeed, the differing balance of homeostatic and damage-associated microglia/macrophages between GF and ABX-treated mice implies that our ABX treatment did not simply imitate GF conditions. This may be due to other “off-target” effects (undetected in our cell culture studies), or perhaps an incomplete depletion of the microbiota, in turn allowing other microorganisms to thrive. Notably, while our ABX regime reduced bacterial DNA by ∼100-fold, other studies have achieved a more extensive depletion (4). Regardless, the lack of an effect on the proportion of myelinated axons at 14 dpl is evidence that remyelination itself is resilient to the changes in inflammation that we provoked using antibiotics.

Interactions between microbiota and regeneration have been observed in other tissues where contact is more direct. Skin wounds heal faster in GF mice, associated with reduced neutrophil accumulation (49), while microbiota-derived LPS was found to dictate regenerative responses in the intestinal crypt (50). Similarly, the regenerative capacity of planaria was diminished by pathogenic shifts in their microbiota (51). However, it seems likely that such interactions become less relevant at anatomically distant sites—for example, the evidence linking liver regeneration to the microbiota is more contentious (52, 53). Outside of a regenerative context, changes in developmental or adaptive myelination in the CNS have previously been linked to the microbiota. GF mice are hypermyelinated specifically in the prefrontal cortex (PFC) (54), while a PFC hypomyelination phenotype could be transferred between different strains of mice by FMT (55). While a role for the microbiota in myelin homeostasis is exciting, the findings of both studies were region-specific and occur in a very different context than remyelination, which is accompanied by tissue damage and a robust inflammatory response.

Although neither our GF nor probiotic study demonstrated a difference in remyelination, in both cases, there was a protective effect of the intervention on demyelination, as seen by increased numbers of oligodendrocytes at time points too early for substantial OPC differentiation to have occurred. This could be explained by the role of the immune system in mediating demyelination as well as remyelination in the lysolecithin and cuprizone models (47, 48, 56, 57). Consistent with this idea, we observed that both of these manipulations of the microbiota were associated with reduced numbers of P2ry12^lo^Clec7a^+^ degeneration-associated microglia/macrophages within the lesion. Similarly, GF mice were resistant to immune-mediated CNS pathology in a model of Parkinson’s disease (46), while changes in the immune response with VSL#3 treatment were associated with white matter preservation in a spinal cord contusion model (44). As the immune system plays a prominent role in demyelination in MS, the majority of previous work on the gut−brain axis has focused on interventions that can limit inflammation and demyelination. For example, in the immune-driven experimental autoimmune encephalomyelitis model, GF or antibiotics-treated mice are resistant to demyelination (19, 58, 59).

In conclusion, we have highlighted the microbiota as a contributing factor to the inflammatory response during CNS remyelination. However, the regenerative responses of OPCs were largely independent of our interventions. The exception to this is following broad-spectrum oral antibiotic treatment, during which antibiotics do not inhibit OPCs directly, but may have other off-target systemic effects. These findings identify high, combined doses of oral antibiotics as a negative influence on OPC responses during remyelination and further our understanding of the interaction between the microbiota and mammalian regeneration.

## Materials and Methods

For full details, see *SI Appendix*, *Supplementary Materials and Methods*.

### Animal Work.

All animal work complied with the requirements and regulations of the United Kingdom Home Office (Project Licenses 70/7715 and 2789) or the European Guidelines for animal welfare with approval from the “Landesamt für Gesundheit und Soziales” (Berlin registration no. G0184/12). Each study also adhered to the respective institutional guidelines, with approval by the local Animal Welfare and Ethical Review Body. The antibiotics study was conducted at Queen’s University Belfast and Charité–Universitätsmedizin Berlin, the germ-free study at the University of East Anglia, and the probiotic study at the University of Cambridge.

### Focal Lysolecithin Lesions in Antibiotics-Treated Mice.

A mixture of antibiotics was administered via the drinking water to 4-mo-old female C57BL/6 mice for 8 wk. The antibiotics used were ampicillin/sulbactam (1.5 g/L), ciprofloxacin (200 mg/L), vancomycin (500 mg/L), metronidazole (1 g/L), and imipenem (250 mg/L)—a regime previously employed to cause depletion of microbes in the gut (4, 60). The fecal transplant group were then orally gavaged with fecal material from control mice, once per day for 5 d, while the antibiotics group continued to receive the antibiotics for the duration of the experiment. The control group were housed in SPF conditions throughout. At age 7 mo, demyelination was initiated by stereotactic injection of 1 μL of lysolecithin (L4129; Sigma-Aldrich) into the thoracic spinal cord ventral white matter.

### Cuprizone Administration to GF Mice.

Male C57BL/6 mice were bred from a GF nucleus colony at the University of East Anglia and maintained in a flexible-film isolator, supplied with sterilized air, food, water, and bedding. The GF group were maintained in these conditions throughout the experiment and compared to age-matched SPF controls. The ex-GF mice were littermates of the GF group and remained in GF conditions until after weaning (4 wk old), at which point they acquired a microbiome by cohousing with the SPF mice. Demyelination was initiated by administration of a 0.2% cuprizone diet (TD.140803; Envigo), which received 50 kGy of γ-irradiation for sterilization. All groups received the cuprizone diet for 5 wk, beginning at age 2 mo, in place of their regular diet and were then returned to regular diet for 3 wk afterward.

### Focal Lysolecithin Lesions in Probiotic-Treated Mice.

The 13-mo-old female C57BL/6 mice were administered a daily dose of 1.35 × 10^9^ colony-forming units (CFU) of VSL#3 (a gift from Janine DeBeer, Ferring Pharmaceuticals, London, United Kingdom), suspended in 100 μL of autoclaved water, by oral gavage for 28 d. These were compared to age-matched control mice, which were instead gavaged daily with 100 μL of water. All groups received a focal injection of lysolecithin as described in *Focal Lysolecithin Lesions in Antibiotics-Treated Mice*.

### Histological Analysis.

Standard techniques for immunohistochemistry and preparation of resin sections were applied, as described previously (61). For a full list of antibodies, please see *SI Appendix*, *Supplementary Materials and Methods*.

### Cell Culture.

Microglia were isolated from 3-mo-old adult C57BL/6 mice using a Magnetic-Activated Cell Sorting (MACS) protocol (Fig. 3*A*), similar to that described previously (62). After 48 h, media was changed to macrophage serum-free medium (Thermo Fisher Scientific), containing the antibiotic treatments (*SI Appendix*, Fig. S2*A*). Following a further 48 h, 10 μg/mL myelin debris was added to each well for 4 h.

OPCs were isolated from P6-8 C57BL/6 mouse pups using a MACS protocol (Fig. 3*D*), similar to that described previously (62). Cultures received growth factors for the first 4 d (PDGFα and FGF2), after which antibiotic treatments (*SI Appendix*, Fig. S2*A*) were introduced for a further 6 d (replaced after 3 d) in the absence of growth factors to allow differentiation. Then 10 μM EdU was applied for 3 h prior to fixation.

Standard immunocytochemistry techniques were applied, as described previously (62). For a full list of antibodies, please see *SI Appendix*, *Supplementary Materials and Methods*.

### Image Analysis.

Cell counts were semiautomated, using a combination of Fiji, CellProfiler, and CellProfiler Analyst software (63). The region of interest (i.e., the lesion area) was manually defined by a blinded observer using a composite image, based on Hoechst^+^ hypercellularity and nonspecific background staining. To quantify the area of a lesion occupied by myelin debris, a threshold determined by background (median) dMBP staining was applied to images in CellProfiler.

To quantify remyelination from toluidine blue-stained resin sections in the probiotic study, slides of the 10 lesions (5 per group) were independently ranked by 2 experienced, blinded investigators (G.A.G. and C.Z.) according to the extent of remyelination. Ranks were based upon the proportion of each lesion with thin myelin sheaths characteristic of remyelination, compared to areas with persistently demyelinated axons. The assigned numerical order (1 to 10) was used for subsequent nonparametric statistical tests. To quantify remyelination from electron microscopy images, the internal and external diameters of myelin sheaths were traced using a freehand selection tool in Fiji. The g-ratio was calculated as the ratio between the diameters of 2 circles with areas equal to the internal and external selections.

### Statistical Analysis.

All statistical analysis was carried out using a Jupyter Notebook with Python 2. In vivo experiments contained the following numbers of biological replicates per group: antibiotics lysolecithin study: *n* = 4 to 6 mice; GF cuprizone study: *n* = 4 to 5 mice; probiotic lysolecithin study: *n* = 3 to 5 mice. These group sizes were chosen based on previous work and were thought to be sufficiently powered to detect meaningful differences in the OPC/inflammatory response to demyelination. For in vivo cell counts, generally 3 to 4 technical replicate sections were counted and averaged per biological replicate. For in vitro cell assays, 3 to 5 technical replicate wells were averaged for each of 4 to 5 biological replicate studies.

Data were tested for normality of residuals (Kolmogorov−Smirnov test) and homogeneity of variance (Levene’s test). Datasets passing both of these criteria were compared by either unpaired Student’s *t* test (if 2 groups), or one-way ANOVA with Tukey honest significant difference (HSD) post hoc tests (if >2 groups). Nonparametric data were compared by Mann−Whitney *U* test (2 groups) or Kruskal−Wallis test with Dunn’s post hoc test (>2 groups). For in vitro assays, treated conditions were compared to control conditions using a paired-samples *t* test with the Holm−Bonferroni correction for multiple comparisons. For all statistical tests, differences were considered significant if *P* < 0.05, and the respective test is described in each figure legend.

In all bar plots in figures, the height of the bar represents the group mean, with an error bar representing the SEM. In vivo data are overlaid with strip plots, in which a gray point represents the value for each individual animal.

### Data Availability Statement.

All data discussed in the paper are available in the main text or *SI Appendix*.

## Supplementary Material

Supplementary File
