# Supplementary material for: The microbiota regulates murine inflammatory responses to toxin-induced CNS demyelination but has minimal impact on remyelination
Source: Proc Natl Acad Sci U S A. 2019 Nov 18;116(50):25311–21. doi: 10.1073/pnas.1905787116 (PMC6911206; doi:10.1073/pnas.1905787116)
Supplement: Supplementary File [file pnas.1905787116.sapp.pdf]

PNAS
www.pnas.org

Supplementary Information for

The microbiota regulates murine inflammatory responses to toxin-induced CNS demyelination but has minimal impact on remyelination

Christopher E McMurran, Alerie Guzman de la Fuente, Rosana Penalva, Ofra Ben Menachem-Zidon, Yvonne Dombrowski, John Falconer, Ginez A Gonzalez, Chao Zhao, Fynn N Krause, Adam MH Young, Julian L Griffin, Clare A Jones, Claire Hollins, Markus M Heimesaat, Denise C Fitzgerald and Robin JM Franklin

Corresponding authors: Denise C Fitzgerald and Robin JM Franklin
Email: d.fitzgerald@qub.ac.uk, rjf1000@cam.ac.uk

This PDF file includes:

Supplementary Materials and Methods
Figures S1 to S7
SI References

www.pnas.org/cgi/doi/10.1073/pnas.1905787116

Supplementary Materials and Methods

Animal work

All animal work complied with the requirements and regulations of the United Kingdom Home Office (Project Licences: 70/7715 and 2789) or the European Guidelines for animal welfare with approval from the "Landesamt für Gesundheit und Soziales" (LAGeSo, Berlin registration number: G0184/12). Each study also adhered to the respective institutional guidelines, with approval by the local Animal Welfare and Ethical Review Body (AWERB). The antibiotics study was conducted at Queen's University Belfast and Charité – Universitätsmedizin Berlin, the germ-free study at the University of East Anglia and the probiotic study at the University of Cambridge. Three to five mice of the same group were housed together in a cage, with *ad libitum* access to water and standard chow diet (other than during cuprizone administration) in a 12-hour light/dark cycle.

Focal lysolecithin lesions in antibiotics-treated mice.

A cocktail of antibiotics was administered via the drinking water to 4-month old female C57BL/6 mice for 8 weeks. The antibiotics used were: ampicillin/sulbactam (1.5g/L), ciprofloxacin (200mg/L), vancomycin (500mg/L), metronidazole (1g/L) and imipenem (250mg/L) – a regime previously employed to cause depletion of microbes in the gut (1, 2). The faecal transplant group were then orally gavaged with faecal material from control mice, once per day for 5 days, whilst the antibiotics group continued to receive the antibiotics for the duration of the experiment. The control group were housed in specific pathogen free (SPF) conditions throughout. Mice were randomly allocated between these three groups at the start of the experiment. At age 7 months, demyelination was initiated by stereotactic injection of 1µl lysolecithin (L4129, Sigma-Aldrich, UK) into the thoracic spinal cord ventral white matter, under isoflurane anaesthesia. At 7 or 14 dpl, mice were intracardially perfused with 4% paraformaldehyde (PFA), the spinal cord was then dissected, post-fixed in cold PFA for 4-6 hours before overnight cryoprotection with 20% sucrose and OCT embedding. Tissue was cut on a cryostat in 12µm thick sections.

Cuprizone administration to germ-free mice.

Male C57BL/6 mice were bred from a GF nucleus colony at the University of East Anglia and maintained in a flexible-film isolator, supplied with sterilised air, food, water and bedding. The GF group were maintained in these conditions throughout the experiment and compared to aged-matched SPF controls. The ex-GF mice were littermates of the GF group and remained in GF conditions until after weaning (4 weeks old), at which point they acquired a microbiome by co-housing with the SPF mice. Animals and isolators were monitored by routine in-house screening using aerobic and anaerobic culture methods, as well as microscopy of faecal smears. Demyelination was initiated by administration of a 0.2% cuprizone diet (TD.140803, Envigo, Huntingdon, UK), which received 50kGy γ-irradiation for sterilisation. All groups received the cuprizone diet for 5 weeks beginning at age 2 months in place of their regular diet and were then returned to regular diet for 3 weeks afterwards. Cuprizone diet was stored sealed at 4°C and replaced every 2-3 days during administration. During the 5-weeks of cuprizone diet, mice were regularly weighed and if substantial weight loss occurred (>15%), the diet was supplemented with small quantities of normal chow, given equally across all three groups. Mice were perfused in the same manner as the antibiotics study either before the initiation of cuprizone (naïve), after 5 weeks cuprizone diet (5w) or after a subsequent 3 weeks of normal diet (5+3w). The brain was retrieved and bisected sagittally, with the left hemisphere post-fixed in PFA for immunohistochemistry (IHC) and the right hemisphere post-fixed in 4% glutaraldehyde for electron microscopy analysis. For IHC, 20µm coronal sections of the left hemisphere were taken from bregma -1 to 1mm.

Focal lysolecithin lesions in probiotic-treated mice.

13-month old female C57BL/6 mice were administered a daily dose of $1.35x10^9$ colony forming units of VSL#3 (a gift from Janine DeBeer, Ferring Pharmaceuticals, London, UK), suspended in 100µl autoclaved water by oral gavage for 28 days. These were compared to age-matched control mice, which were instead gavaged daily with 100µl water. Randomly allocated control and probiotic-treated mice were housed in separate racks necessitating a change of gloves between handling, to avoid cross-contamination. Following this treatment, all groups received a focal injection of lysolecithin as described in the antibiotics study. Gavage treatments were recommenced from two days after this surgery and mice were sacrificed by perfusion-fixation at 5 or 14 dpl with 4% PFA or at 21 dpl with 4% glutaraldehyde.

Immunohistochemistry

Cryosections on glass slides were brought to room temperature (RT) and rehydrated with PBS, prior to an antigen retrieval step, consisting of a 10-minute RT incubation with boiling antigen retrieval solution (Dako, Ely, UK). Slides were washed three times with PBS and blocked for 1 hour with 5% Normal Donkey Serum (NDS, Sigma-Aldrich) and 0.3% triton in PBS. Sections were then incubated with primary antibodies diluted in blocking solution in a humidity chamber overnight at 4°C. Primary antibodies: goat anti-Arg1 1:200 (sc-18351, Santa Cruz, Dallas, TX), mouse anti-CC1 1:100 (OP80, Calbiochem, San Diego, CA), rabbit anti-CD68 1:500 (ab125212, Abcam, Cambridge, UK), rat anti-CD68 1:200 (MCA1957, Serotec, Kiddlington, UK), rat anti-Clec7a [Dectin-1] 1:200 (mabg-mdect, InvivoGen), rabbit anti-dMBP 1:500 (AB5864, Millipore, Burlington, MA), rabbit anti-Iba1 1:500 (019-10741, Wako, Osaka, Japan), rabbit anti-iNOS 1:200 (ab136918, Abcam), rabbit anti-Ki67 1:300 (ab15580, Abcam), rat anti-MBP 1:400 (MAB384, Millipore), rat anti-MHC class II 1:500 (14-5321-82, eBioscience, Thermo Fisher Scientific, San Diego, CA), goat anti-mannose receptor 1:500 (AF2535, R&D Systems, Minneapolis, MN), rabbit anti-Olig2 1:500 (AB9610, Millipore), rabbit anti-neurofilament 1:400 (ab8135, abcam), rabbit anti-P2ry12 1:200 (a gift from Oleg Butovsky, Harvard Medical School), goat anti-Sox10 1:200 (sc-17342, Santa Cruz), rabbit anti-Tmem119 [28-3] 1:400 (ab209064, Abcam). Slides were washed three times with PBS and incubated with relevant fluorophore-conjugated secondary antibodies (all Invitrogen, Thermo Fisher Scientific) diluted 1:500 in blocking solution for 2 hours at RT and protected from light. Secondary antibodies: Alexa 488 donkey anti-mouse (A21202), Alexa 488 donkey anti-rabbit (A21206), Alexa 488 donkey anti-rat (A21208), Alexa 568 donkey anti-goat (A11057), Alexa 568 donkey anti-mouse (A10037), Alexa 568 donkey anti-rabbit (A10042), Alexa 647 donkey anti-mouse (A31571), Alexa 647 donkey anti-rabbit (A31573). Nucleic acids were stained by 10 minutes incubation with 1µg/ml Hoechst. After three more PBS washes, sections were washed with water then mounted using Fluoromount-G (Southern Biotech, Birmingham, AL) with glass coverslips (VWR, Lutterworth, UK). Where mouse-derived primary antibodies were to be used on mouse tissue, a mouse-on-mouse blocking kit (Vektor, Peterborough, UK), was used according the manufacturer's instructions.

Fluorescently immunolabelled sections were imaged using a Leica-SP5 confocal microscope with LAS software (Leica Microsystems, Wetzlar, Germany). Depending on the staining, either Z-stacks spanning the section widths were obtained and combined, or a single planar image was taken, and either a 20x or 40x lens was used.

Histological analysis of remyelination

To produce semi-thin resin sections for toluidine blue staining, glutaraldehyde-fixed tissue was dissected into pieces of maximum 1 mm thickness and stained with 2% osmium tetroxide overnight at 4°C. Samples were then processed into resin (TAAB Laboratories Equipment Ltd., Aldermaston, UK), after which 0.75µm sections were cut using a microtome (Leica RM 2065) and stained with 1% toluidine blue.

These samples were further analysed by electron microscopy. 0.75µm sections were stained with aqueous 4% uranylacetate and lead citrate and visualised on a Tecnai G2 80-200keV transmission electron microscope. A minimum of five micrographs per animal were captured at 5000x from the splenium of the corpus callosum, close to the midline.

Microglia isolation and culture

Microglia were isolated from 3-month old adult C57BL/6 mice using a Magnetic-Activated Cell Sorting (MACS) protocol (**Fig. 3A**). Mice were euthanised by CO_2 overdose and posterior cervical dislocation, and each brain provided cells for one replicate. Tissue was diced into small pieces and incubated at 37°C for 30 minutes in a dissociation solution, consisting of 34 U/ml papain (Worthington, Lakewood, NJ) and 20 µg/ml DNAse (Gibco, Thermo Fisher Scientific) in HALF (Hibernate-A equivalent, made in house). After this, tissue was triturated with a fire-polished glass pipette, passed through a 70µm cell strainer (Millipore) and centrifuged for 20 minutes at 800g in 22.5% Percoll (GE Healthcare, Little Chalfont, UK). The pellet, containing single cells, was labelled with magnetic bead-conjugated antibodies for CD11b (Miltenyi Biotech, Woking, UK), and $CD11b^+$ microglia were eluted by MACS according the manufacturer's instructions. Microglia were cultured at $2x10^4$ cells per well of a poly-d-lysine-coated 96-well microplate (Corning, NY), in DMEM/F12 (Gibco) supplemented with 10% foetal bovine serum (FBS, Biosera, Heathfield, UK), 2%

B27, 500µM N-acetylcysteine and 1% penicillin-streptomycin. After 48 hours, media was changed to macrophage serum-free medium (Thermo Fisher Scientific), containing the antibiotic treatments. Following a further 48 hours, 10µg/ml myelin debris was added to each well for 4 hours. Myelin debris had been isolated from 2 to 3-month old C57BL/6 mice by discontinuous sucrose gradient centrifugation, as previously described (3, 4). After this incubation, un-internalised debris was removed by washing with cold PBS and cells were fixed with 4% PFA.

OPC isolation and culture

OPCs were isolated from P6-8 C57BL/6 mouse pups using a MACS protocol (**Fig. 3D**), with pups euthanised by overdose of Pentoject (Animalcare Ltd., Hull, UK) and brains from 2-3 pups pooled per replicate. The isolation was otherwise identical to the microglia protocol until the labelling stage, at which point cells were instead incubated with 1:1000 anti-A2B5 (Millipore) and then a magnetic bead-coupled goat-anti-mouse-IgM antibody (Miltenyi Biotech), prior to MACS. $A2B5^{+}$ OPCs were cultured at 5000 cells per well of a poly-d-lysine and laminin-coated 96-well microplate, in DMEM/F12 (Gibco) supplemented with 5µg/ml insulin (Gibco), 1x Trace Elements B (Corning), 100µg/ml apo-transferrin, 16.1µg/ml putrescine, 40ng/ml sodium selenite, 60ng/ml progesterone, 60µg/ml N-acetylcysteine and 1% penicillin-streptomycin, 5µM forskolin and 1ng/ml biotin (all Sigma-Aldrich unless otherwise indicated). Cultures received growth factors for the first 4 days (10ng/ml/day of platelet-derived growth factor α (PDGFα) and fibroblast growth factor 2 (FGF2)), after which antibiotic treatments were introduced for a further six days (replaced after 3 days) in the absence of growth factors to allow differentiation. Cells were then incubated with 10µM 5-ethynyl-2'-deoxyuridine (EdU) for 3 hours prior to fixation with 4% PFA.

Antibiotic treatments *in vitro*

To apply antibiotic treatments (all Sigma-Aldrich) at doses approximating those of *in vivo* exposure, steady state plasma concentrations ($C_{SS(P)}$) were estimated for each antibiotic administered to mice in their drinking water (Fig. S2A). These estimations were based on literature values of oral bioavailability (F), clearance (CL), and area-under-the-curve ratio of cerebrospinal fluid (CSF) to plasma (AUC_{CSF}/AUC_P), for ampicillin and sulbactam (5–7), ciprofloxacin (8–10) and metronidazole (10–12). Daily water consumption was assumed to be 4 ml/day/mouse.

Immunocytochemistry

Fixed cells in 96-well plates were blocked for 1 hour with 5% NDS and 0.1% triton in PBS. For the cell proliferation assay, EdU was labelled at this point using the Click-iT EdU Alexa Fluor 647 Imaging Kit (Thermo Fisher Scientific). Cells were then incubated with primary antibodies diluted in blocking solution overnight at 4°C. Primary antibodies: mouse anti-CNPase 1:500 (C5922, Sigma-Aldrich), rabbit anti-Iba1 1:1000 (019-10741, Wako), rat anti-MBP 1:500 (MAB384, Millipore), rabbit anti-Olig2 1:500 (AB9610, Millipore). Cells were then washed three times with PBS and incubated with relevant fluorophore-conjugated secondary antibodies (see "Immunohistochemistry") diluted in blocking solution for 1 hour at RT and protected from light. Nucleic acids were stained by 10 minutes incubation with 1µg/ml Hoechst, followed by three more PBS washes. Images were acquired automatically using a Cell Insight CX5 (Thermo Fisher Scientific).

Faecal polymerase chain reaction (PCR)

To determine the microbial load of antibiotics-treated mice, DNA from faecal pellets was extracted as described previously (1), quantified using QuantiT PicoGreen reagent (Invitrogen) and adjusted to 1 ng/µl. Copy numbers of the 16S rRNA gene were quantified by quantitative RT-PCR using generic eubacterial primers (Tib MolBiol, Berlin, Germany), and expressed per ng of total DNA.

To confirm microbial status of GF and ex-GF mice, DNA from faecal pellets was isolated using a QIAamp DNA Stool Mini Kit (Qiagen, Hilden, Germany) following the manufacturer's instructions. 1µl eluted DNA was added to 24µl PCR SuperMix (Thermo Fisher Scientific) with 200nM *UniF/R* primer pairs (13), which recognise a 147bp conserved region of bacterial 16S rRNA. The conditions for the PCR were 95°C for 5 minutes, then 25 cycles of 95°C for 30s, 52°C for 30s and 72°C for 45s, and finally 72°C for 7 minutes. The PCR products were then separated on a 1% agarose gel and visualised under UV light.

Detection of faecal / serum metabolites using GC-MS

Fresh faecal pellets were collected and stored in sterile Eppendorf tubes at -80°C. Serum samples were obtained from 100-200µl left ventricular blood collected prior to perfusion. The blood samples were incubated in sterile Eppendorf tubes for 1 hour at RT to allow coagulation, then separated by centrifugation for 15 minutes at 1500g. Serum was collected and stored at -80°C.

Short chain fatty acids (SCFAs) were extracted using a modified Bligh and Dyer method (14). In short, 15-25mg of faeces or 20µl of serum was transferred to a pre-chilled plastic tube and extracted with ice-cold 2:2:1 methanol:chloroform:water containing internal standard, following vigorous mixing, sonication and centrifugation (16,000g, 20 minutes). For serum samples, 100µl of the aqueous phase was transferred to pre-chilled glass tubes. Faecal samples were extracted twice, and a combined total of 300µl aqueous phase was transferred to pre-chilled glass tubes. Aqueous phases were dried down under nitrogen at 4°C, and derivatised as described previously (15).

SCFAs were measured by gas chromatography-mass spectrometry (GC-MS), on a Trace GC Ultra coupled to a Trace DSQ II mass spectrometer (Thermo Fisher Scientific). Derivatised samples were diluted 1:1 with hexane, and 2µl was injected onto a 50m x 0.25mm (5% phenyl-arylene, 95% dimethylsiloxane) column with a 0.25µm ZB-5MS stationary phase (Phenomenex, Macclesfield, UK). Full-scan spectra were collected at three scans per second over a range of 50 to 650 *m/z*. Data processing was carried out using *Xcalibur* (version 2.2, Thermo Fisher Scientific), and peaks were assigned based on the *National Institute of Standards and Technology* (USA) library. All solvents were of HPLC-grade or higher.

Image analysis
Cell counts were semi-automated, using a combination of *Fiji*, *CellProfiler* and *CellProfiler Analyst* software (16). *Fiji* was used to create maximum projections of Z-stacks acquired by confocal microscopy. The region of interest (ROI i.e. the lesion area) was manually defined by a blinded observer using a composite image, based on Hoechst$^+$ hypercellularity and non-specific background staining. Individual channels were then extracted and imported into *CellProfiler*, where the nuclear channel (Hoechst) was cropped to the pre-defined ROI, and nuclei were identified as primary objects. Other channels were normalised to the median background intensity to correct for variability in staining. A number of intensity features were measured for the nuclear and peri-nuclear regions of each cell in every channel, and this data was exported as a master database file. In *CellProfiler Analyst*, a training set of >50 cells was specified per group and used to train a classifier based on the feature set. For CD68 and P2ry12, which are expressed to different degrees by cells within the lesion, these were grouped into "high" and "low" classes of expression, and examples of cells from these training sets are provided (**Fig. S1M, N**). The training data was increased until the classifier gave consistently comparable results to manual counting in sample images. Finally, "per lesion" cell counts were extracted for each image using this classifier.

To quantify the area of a lesion occupied by myelin debris, images of tissue stained for dMBP were imported into a *CellProfiler* pipeline, which applied a threshold to each image determined by the background (median) staining. The area of the image above this threshold was considered positive for myelin debris and was expressed as a fraction of the total lesion area. For morphological analysis of microglia, Iba1$^+$ microglia were identified in *CellProfiler Analyst*, and this population were then further analysed within *CellProfiler* to quantify morphological features of each cell's Iba1$^+$ mask and skeleton.

To quantify remyelination from toluidine blue-stained resin sections in the probiotic study, slides of the 10 lesions (5 per group) were independently ranked by two experienced, blinded investigators (GG and CZ) according to the extent of remyelination. Ranks were based upon the proportion of each lesion with thin myelin sheaths characteristic of remyelination, compared to areas with persistently demyelinated axons. The assigned numerical order (1-10) was used for subsequent non-parametric statistical tests. To quantify remyelination from electron microscopy images, only axons contained entirely within each field were counted. For these axons, the internal and external diameter of the myelin sheath were then traced using a freehand selection tool in *Fiji*. The g-ratio was calculated as the ratio between the diameters of two circles with areas equal to the internal and external selections respectively. Axons with a circularity <0.7 or aspect ratio >2.5 were excluded from further analysis.

Statistical analysis

All statistical analysis was carried out using a *Jupyter* Notebook with *Python 2*. *In vivo* experiments contained the following numbers of biological replicates per group: antibiotics lysolecithin study: n = 4-6 mice, germ-free cuprizone study: n = 4-5 mice, probiotic lysolecithin study: n = 3-5 mice. These group sizes were chosen based on previous work and were thought to be sufficiently powered to detect meaningful differences in the OPC / inflammatory response to demyelination. For *in vivo* cell counts, generally 3-4 technical replicate sections were counted and averaged per biological replicate. For *in vitro* cell assays, 3-5 technical replicate wells were averaged for each of 4-5 biological replicate studies.

Data was tested for normality of residuals (Kolmogorov-Smirnov test) and homogeneity of variance (Levene's test). Data sets passing both of these criteria were compared by either unpaired Student's *t*-test (if 2 groups), or one-way ANOVA with Tukey HSD *post hoc* tests (if >2 groups). Non-parametric data was compared by Mann-Whitney *U* test (2 groups) or Kruskal-Wallis test with Dunn's *post hoc* test (>2 groups). For *in vitro* assays, treated conditions were compared to control conditions using a paired-samples *t*-test with the Holm-Bonferroni correction for multiple comparisons. For ranking analysis of remyelination, groups were compared using the Mann-Whitney *U* test. For all statistical tests, differences were considered significant if $p<0.05$, and the respective test is described in each figure legend.

In all bar plots, the height of the bar represents the group mean, with an error bar representing the standard error of the mean (SEM). *In vivo* data are overlaid with strip plots, in which a grey point represents the value for each individual animal.

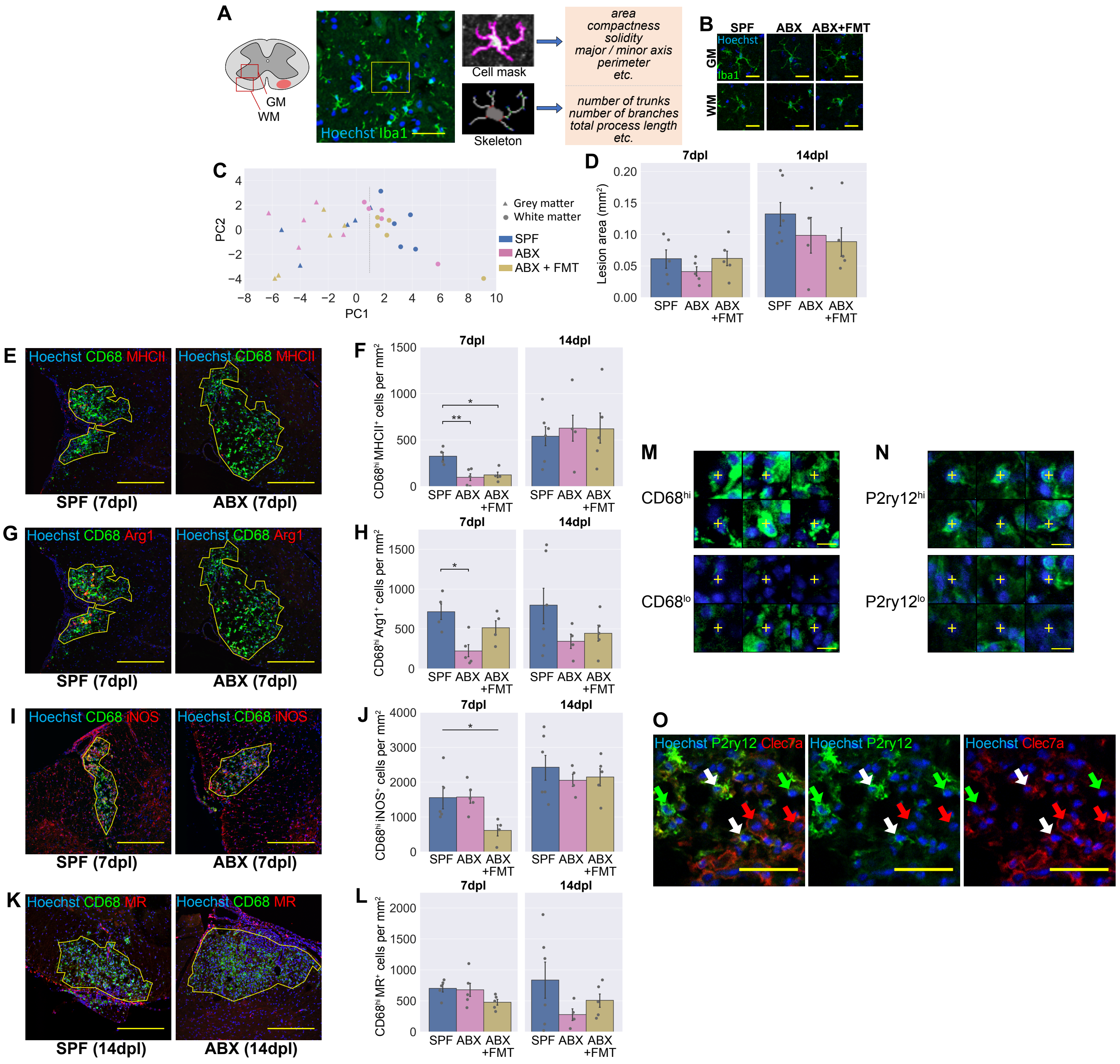

Fig. S1. Morphometric analysis of microglia in unlesioned white matter and other features of the inflammatory response to demyelination in ABX-treated mice.
(**A**) Protocol for morphometric analysis: spinal cord sections were stained for Iba1, and images taken in the ventral horn grey matter and ventrolateral white matter, contralateral to the lesion. Microglia (Iba1$^+$) were identified using *CellProfiler* software and a number of morphological features were extracted.
(**B**) Representative images of Iba1$^+$ microglia from the grey (GM) and white matter (WM) of each experimental group.
(**C**) Plot of principal components 1 and 2 from the morphology dataset shows clustering of grey versus white matter microglia (dotted line), but no clear differences between experimental groups.
(**D**) Following demyelination, lesion area was not different between groups.
(**E-L**) Representative images and densities within lesions of activated microglia / infiltrating macrophages co-expressing high levels of CD68 with major histocompatibility complex class II (MHC II, **E-F**), arginase-1 (Arg1, **G-H**), inducible nitric oxide synthetase (iNOS, **I-J**) or mannose receptor (MR, **K-L**).
(**M-N**) Representative images of CD68^{hi} and CD68^{lo} cells (**M**) and of P2ry12^{hi} and P2ry12^{lo} cells (**N**).
(**O**) Representative image of a 5dpl lesion demonstrating examples of P2ry12^{hi}Clec7a$^-$ cells (green arrows), P2ry12^{lo}Clec7a$^+$ cells (red arrows), as well as cells with substantial expression of both markers (white arrows).
Scale bars: (**A, O**) = 50μm, (**B**) = 20μm, (**E, G, I, K**) = 250μm, (M,N) = 10μm. Error bars show mean ± SEM; *p<0.05, **p<0.01; one-way ANOVA with Tukey HSD *post hoc* test, n=4-6 mice.

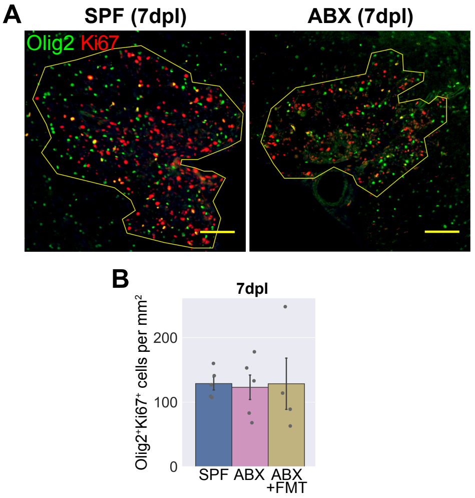

Fig. S2. ABX treatment does not affect OPC proliferation in lesions.
(**A-B**) Representative images (**A**) and density (**B**) of Olig2^{+}Ki67^{+} proliferating OPCs within lesions. Scale bars (**A**) = 100μm. Error bars show mean ± SEM, n=4-5 mice.

A

$$C_{ss} = \frac{F \times \text{dose rate}}{CL}$$

Drug	Orally absorbed?	Drinking water conc. (mg/l)	Dose rate (mg/day)	F	CL (ml/min)	AUC_{CSF}/AUC_P	$C_{ss(P)}$ (ng/ml)	$C_{ss(CNS)}$ (ng/ml)	In vitro dose (ng/ml) ++	+
Ampicillin	✓	1000	4.0	0.23	1.8	0.39*	360	140	150	15
Sulbactam	✓	500	2.0	0.19	3.9	0.34*	68	23	25	2.5
Ciprofloxacin	✓	200	0.8	0.7*	0.81	0.6*	480	290	300	30
Metronidazole	✓	1000	4.0	0.95*	0.068	0.87*	40000	34000	35000	3500
Imipenem	✗							0		
Vancomycin	✗							0		

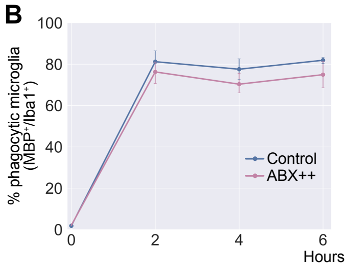

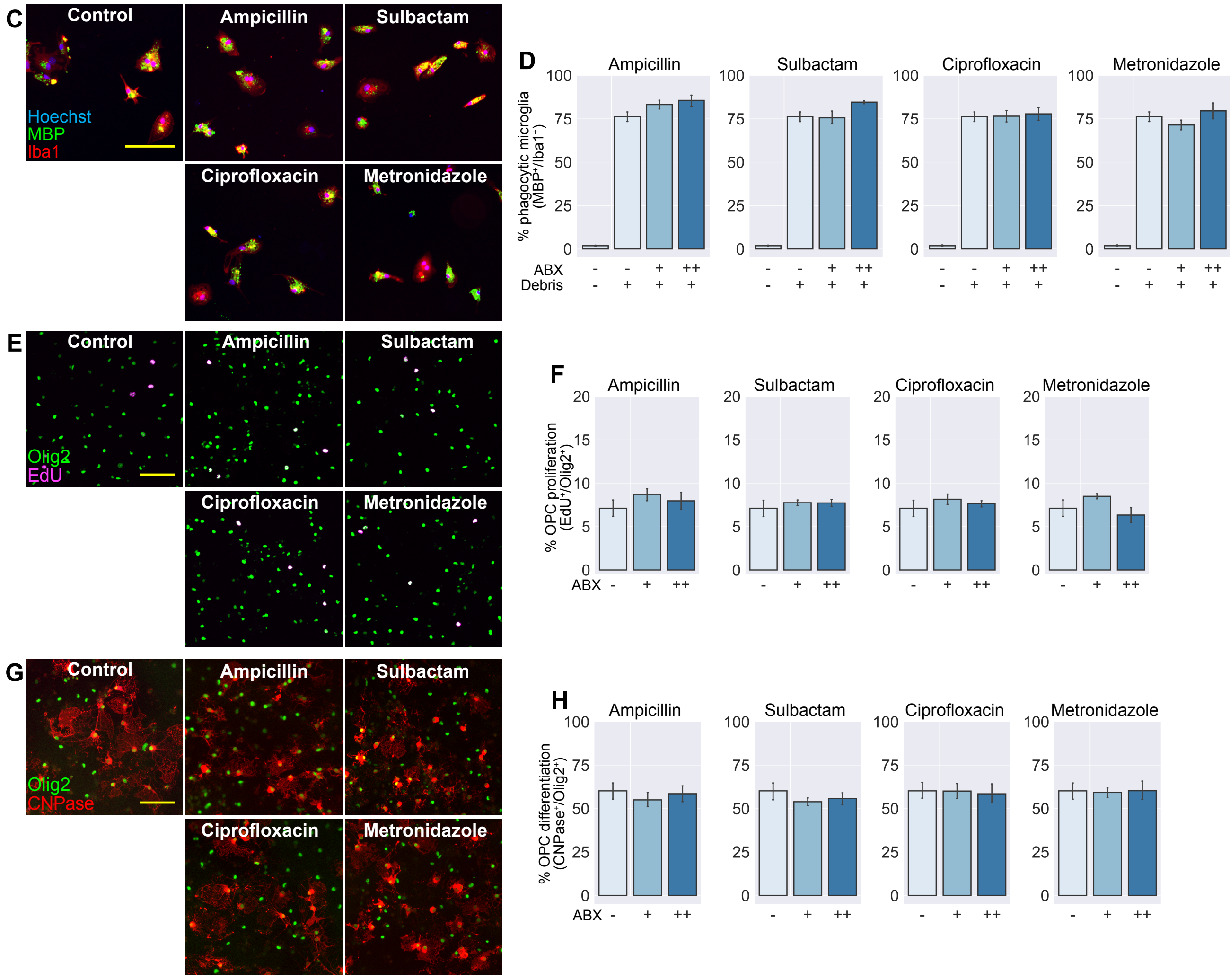

Fig. S3. Further data from *in vitro* antibiotics experiments.
(**A**) CNS concentrations ($C_{SS(CNS)}$) of ABX were estimated using literature values for their bioavailability (*F*), clearance (*CL*) and CNS penetration (AUC_{CSF}/AUC_P). Data from **SI References** 5-12; * denotes values extrapolated from human studies.
(**B**) Timecourse study of myelin phagocytosis over 6 hours showed no difference in the presence of combined ABX treatment at estimated CNS concentrations (++) compared to control conditions.
(**C-D**) Myelin uptake was quantified for each antibiotic individually at its estimated CNS concentration (++) and a 10% dose (+).
(**E-H**) OPC proliferation (**E-F**) and differentiation (**G-H**) were quantified for each antibiotic individually at its estimated CNS concentration (++) and a 10% dose (+).
Scale bars (**C, E, G**) = 100μm. Error bars show mean ± SEM; (**B**) two-way ANOVA, (**D, F, H**) paired-samples *t*-test with Holm-Bonferroni correction, n=4-5 separate experiments.

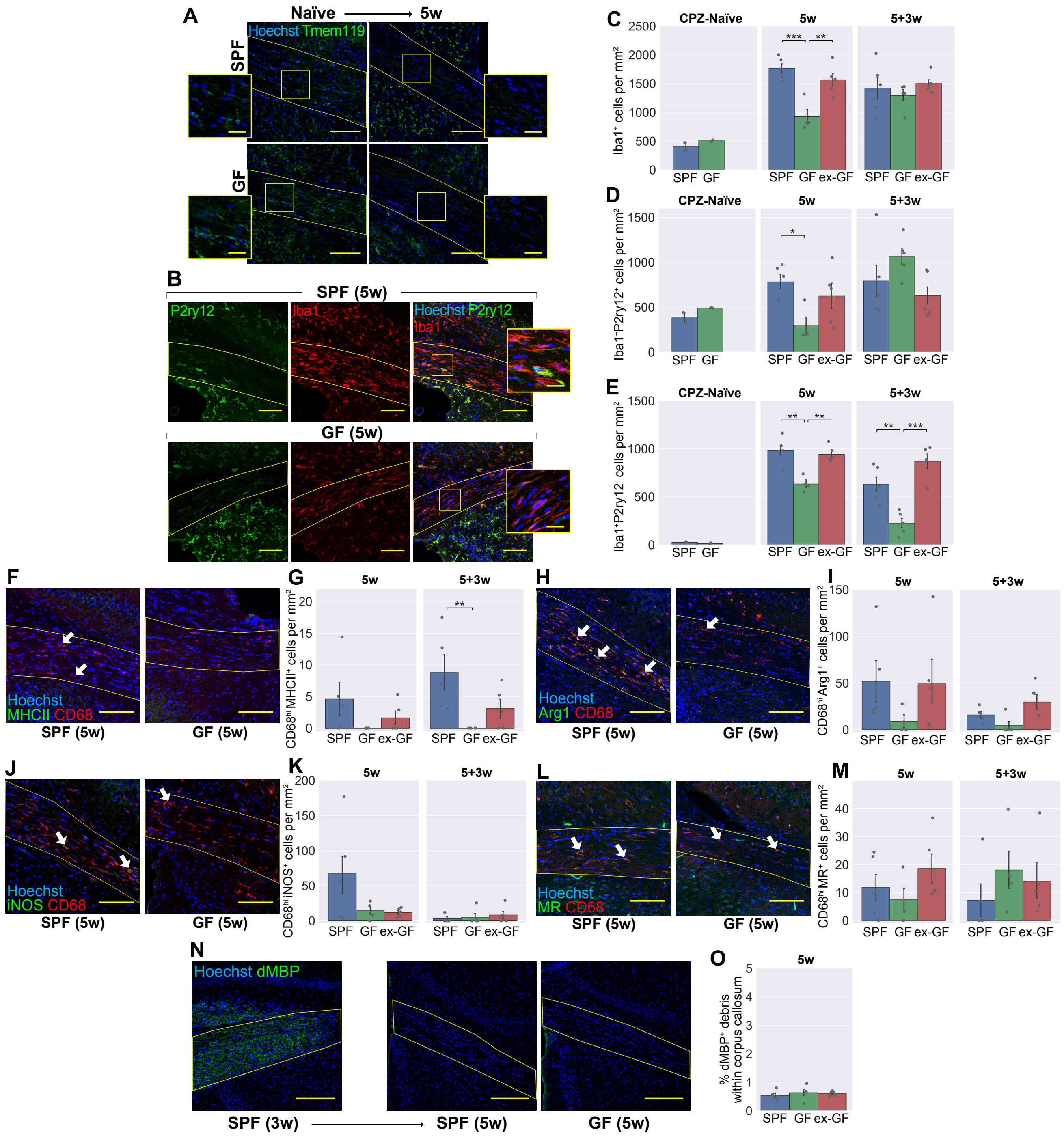

Fig. S4. Further data on the inflammatory response to cuprizone in germ-free mice.
(**A**) Representative images demonstrating low expression of Tmem119 in the corpus callosum (yellow line) following cuprizone exposure in both SPF and GF mice.
(**B**) Representative images of Iba1^{+}P2ry12^{+} microglia and Iba1^{+}P2ry12^{-} monocyte-derived macrophages within the corpus callosum after 5 weeks of cuprizone exposure. Insets are a 3x magnification of the boxed regions.
(**C-E**) Densities of total Iba1^{+} (**C**), Iba1^{+}P2ry12^{+} (**D**) and Iba1^{+}P2ry12^{-} (**E**) cells within the corpus callosum.
(**F-M**) Representative images and densities within the corpus callosum of activated microglia / infiltrating macrophages co-expressing high levels of CD68 with MHC II (**F-G**), Arg1 (**H-I**), iNOS (**J-K**) or mannose receptor (**L-M**).
(**N**) After 3 weeks of cuprizone treatment (in a SPF mouse) there was substantial accumulation of dMBP^{+} myelin debris, which was almost entirely cleared by 5 weeks in both SPF and GF groups.
(**O**) Quantification of the remaining dMBP^{+} myelin debris after 5 weeks of cuprizone treatment.
Scale bars: (**A, B**) = 100µm [inset scale bars = 25µm], (**F, H, J, L**) = 100µm, (**N**) = 200µm. Arrow heads (**F, H, J, L**) show representative double-positive cells. Error bars show mean ± SEM; *p<0.05, *p<0.01, ***p<0.001; (**C-E, I, K, M, O**) one-way ANOVA, (**G**) Kruskal-Wallis with Dunn's *post hoc* test, n=4-5 mice.

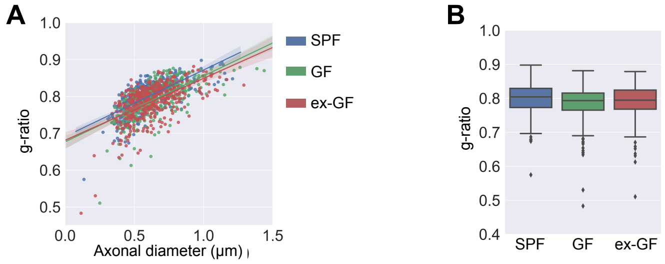

Fig. S5. G-ratio analysis of remyelination in germ-free mice.
(**A**) g-ratios of all axons analysed, expressed as a function of axonal diameter with a regression line for each group. (**B**) Boxplot of g-ratios separated by experimental group.
Shaded areas (**A**) show 95% confidence interval for each line. Outliers (**B**) are more than 1.5 times the interquartile range (IQR) beyond the boundaries of the IQR. SPF n=371 axons; GF n=375 axons; ex-GF n=423 axons.

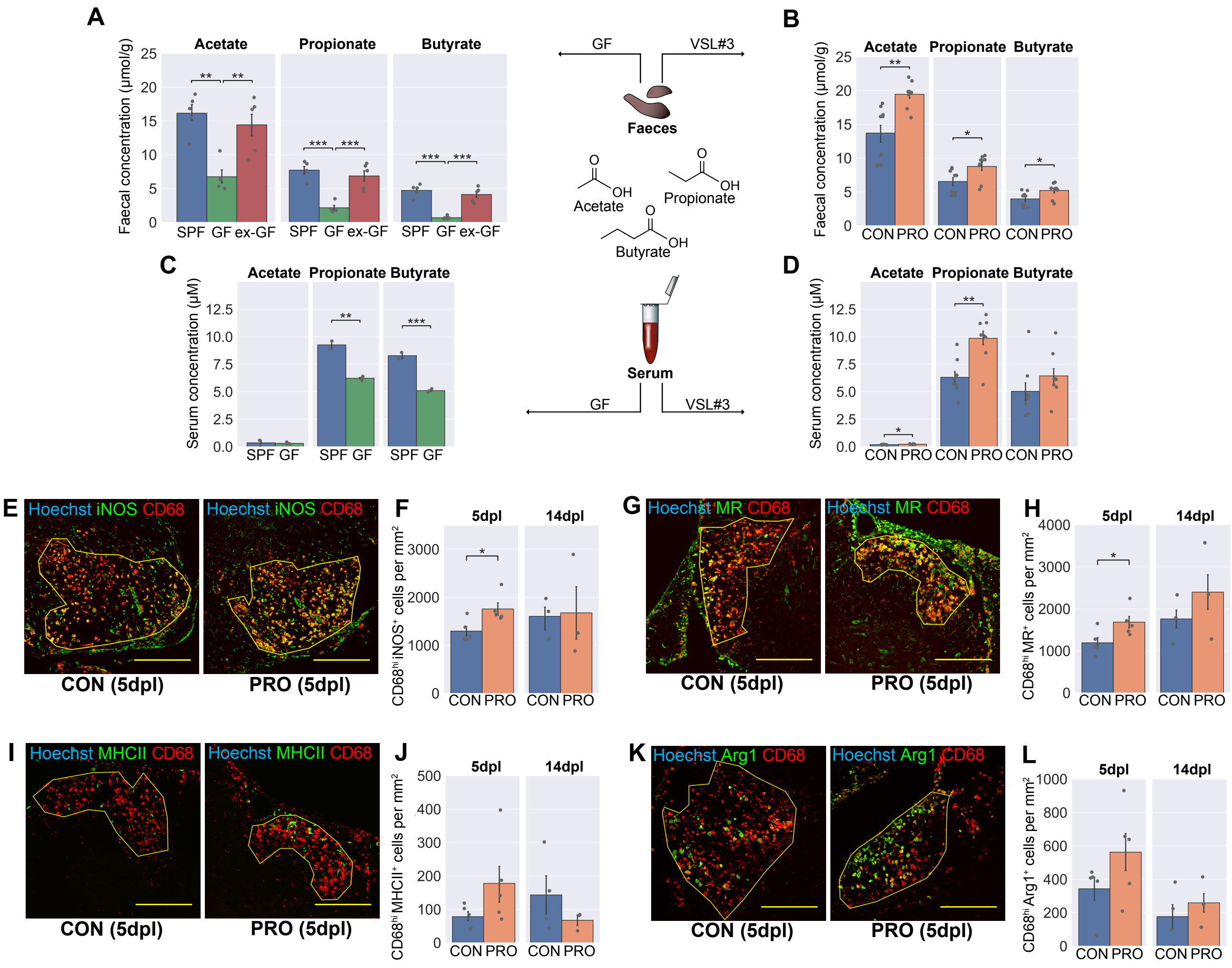

Fig. S6. Additional effects of VSL#3 treatment on SCFAs and inflammation.
(**A-D**) Quantification by gas chromatography-mass spectroscopy (GC-MS) of SCFAs in the faeces of GF (**A**) and VSL#3-treated mice (**B**), and in the serum of GF (**C**) and VSL#3-treated mice (**D**).
(**E-L**) Representative images and densities within the lesions of activated microglia / infiltrating macrophages co-expressing high levels of CD68 with iNOS (**E-F**), mannose receptor (**G-H**), MHC II (**I-J**) or Arg1 (**K-L**).
Scale bars (**E, G, I, K**) = 250µm. Error bars show mean ± SEM; *p<0.05, *p<0.01, ***p<0.001; (**A**) one-way ANOVA with Tukey HSD *post hoc* test n=4-5 mice; (**B, D**) Student's *t*-test, n=8 mice; (**C**) Student's *t*-test, n=2-5 mice; (**F, H, J, L**) Student's *t*-test, n=3-5 mice.

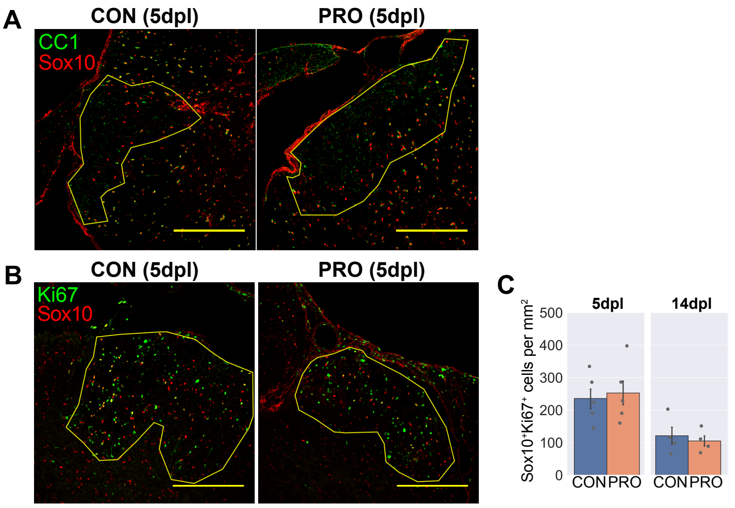

Fig. S7. Additional effects of VSL#3 treatment on OPCs and oligodendrocytes.
(**A**) Representative images of Sox10$^+$CC1$^+$ oligodendrocytes within lesions at 5dpl.
(**B-C**) Representative images (**B**) and density (**C**) of Sox10$^+$Ki67$^+$ proliferating OPCs within lesions. Scale bars (**A, B**) = 250µm. Error bars show mean ± SEM, n=3-5 mice.

SI References

1. L. Möhle et al., Ly6Chi monocytes provide a link between antibiotic-induced changes in gut microbiota and adult hippocampal neurogenesis. *Cell. Rep.* **15**, 1945–1956 (2016).
2. M. M. Heimesaat et al., Gram-negative bacteria aggravate murine small intestinal Th1-type immunopathology following oral infection with Toxoplasma gondii. *J. Immunol.* **177**, 8785–8795 (2006).
3. M. R. Kotter, W.-W. Li, C. Zhao, R. J. M. Franklin, Myelin impairs CNS remyelination by inhibiting oligodendrocyte precursor cell differentiation. *J. Neurosci.* **26**, 328–32 (2006).
4. M. S. Natrajan et al., Retinoid X receptor activation reverses age-related deficiencies in myelin debris phagocytosis and remyelination. *Brain* **138**, 3581–3597 (2015).
5. A. R. English, D. Girard, S. L. Haskell, Pharmacokinetics of sultamicillin in mice, rats, and dogs. *Antimicrob. Agents Chemother.* **25**, 599–602 (1984).
6. G. Foulds et al., Penetration of sulbactam and ampicillin into cerebrospinal fluid of infants and young children with meningitis. *Antimicrob. Agents Chemother.* **31**, 1703–1705 (1987).
7. C. X. Liu, J. R. Wang, Y. L. Lu, Pharmacokinetics of sulbactam and ampicillin in mice and in dogs. *Acta Pharm. Sin.* **25**, 406–411 (1990).
8. G. L. Drusano et al., Absolute oral bioavailability of ciprofloxacin. *Antimicrob. Agents Chemother.* **30**, 444–446 (1986).
9. E. Vallee, E. Azoulay-Dupuis, J. Bauchet, J. J. Pocidalo, Kinetic disposition of temafloxacin and ciprofloxacin in a murine model of pneumococcal pneumonia. Relevance for drug efficacy. *J. Pharmacol. Exp. Ther.* **262**, 1203–1208 (1992).
10. R. Nau, F. Sorgel, H. Eiffert, Penetration of drugs through the blood-cerebrospinal fluid/blood-brain barrier for treatment of central nervous system infections. *Clin. Microbiol. Rev.* **23**, 858–883 (2010).
11. I. I. Al-Dabagh, F.K. Mohammad, Pharmacokinetics and distribution of metronidazole administered intraperitoneally in mice. *Pharmacologyonline* **3**, 858–863 (2008).
12. A. H. Lau et al., Clinical pharmacokinetics of metronidazole and other nitroimidazole anti-infectives. *Clin. Pharmacokinet.* **23**, 328–364 (1992).
13. C. D. Packey et al., Molecular detection of bacterial contamination in gnotobiotic rodent units. *Gut Microbes* **4**, 361–370 (2013).
14. E. Bligh, W. Dyer, A rapid method of total lipid extraction and purification. *Can. J. Biochem. Physiol.* **37**, 911–7 (1959).
15. J. Gullberg, P. Jonsson, A. Nordström, M. Sjöström, T. Moritz, Design of experiments: An efficient strategy to identify factors influencing extraction and derivatization of Arabidopsis thaliana samples in metabolomic studies with gas chromatography/mass spectrometry. *Anal. Biochem.* **331**, 283–295 (2004).
16. A. E. Carpenter et al., CellProfiler: image analysis software for identifying and quantifying cell phenotypes. *Genome Biol.* **7**, R100 (2006).
